# Global research trends on platelet-rich plasma for tendon and ligament injuries from the past two decades: A bibliometric and visualized study

**DOI:** 10.3389/fsurg.2023.1113491

**Published:** 2023-02-10

**Authors:** Jie Xu, Wanli Du, Xiali Xue, Meng Chen, Wenqi Zhou, Xiaobing Luo

**Affiliations:** ^1^ Department of Sports Medicine, Sichuan Provincial Orthopedics Hospital, Chengdu, China; ^2^ Department of Cervicodynia/Omalgia/Lumbago/Sciatica 2, Sichuan Provincial Orthopedics Hospital, Chengdu, China; ^3^ Institute of Sports Medicine and Health, Chengdu Sport University, Chengdu, China; ^4^ Department of Emergency Medicine, Nanchong Hospital of Traditional Chinese Medicine, Nanchong, China

**Keywords:** PRP, bibliometric analysis, tendinopathy, ligaments, citespace, visual analysis

## Abstract

**Background:**

In sports medicine, tendon and ligament injuries are the most prevalent conditions, and with the booming of sports competition, the incidence of sports injuries is gradually increasing, investigating more potent therapeutic options is therefore becoming increasingly crucial. Platelet-rich plasma therapy has gained popularity as an effective and secure treatment in recent years. Currently, a faceted systematic and clear visual analysis is lacking in this research area.

**Methods:**

The literature related to using platelet-rich plasma to treat ligament and tendon injuries from 2003 to 2022 in the core dataset of the Web of Science database was collected and analyzed visually using Citespace 6.1 software. Research hotspots and development trends were analyzed in terms of high-impact countries or regions, authors, research institutions, keywords, and cited literature.

**Results:**

The literature comprised a total of 1,827 articles. The annual publication volume of relevant literature has demonstrated a significant development tendency as the field of platelet-rich plasma research for tendon and ligament injuries has heated up in recent years. With 678 papers, the United States came in top place, followed by China with 187 papers. Hosp Special Surg ranked first with 56 papers. The hot research topics analyzed by keywords were tennis elbow, anterior cruciate ligament, rotator cuff repair, achilles tendon, mesenchymal stem cells, guided tissue regeneration, network meta analysis, chronic patellar tendinopathy, and follow up.

**Conclusion:**

Analysis of the research literature over the past 20 years shows that the United States and China will continue to dominate in terms of volume of publications based on annual volume and trends, with some collaboration among high-impact authors and further collaboration still needed in different countries and institutions. Platelet-rich plasma is widely used in the treatment of tendon ligament injuries. Its clinical efficacy is influenced by a number of factors, the main ones being the inconsistency in the preparation and composition of platelet-rich plasma and its related preparations, and the differences in efficacy due to different activation methods of platelet-rich plasma, as well as factors such as injection time, injection site, administration method, number of administrations, acidity and evaluation methods, In addition, the applicability to different injury diseases remains controversial. In recent years, the molecular biology of platelet-rich plasma for tendon ligament therapy has received increasing attention.

## 1. Introduction

In sports medicine, rehabilitation medicine, and orthopedics, tendon and ligament injuries are the most common soft tissue injuries and are most prevalent in athletes, sports enthusiasts, and other people involved in high-load weight-bearing activities (1). It is the most common soft tissue injury, and it is highly prevalent in athletes, sports enthusiasts, and other people who are involved in high load bearing activities. Approximately 30% of current clinical consultations for musculoskeletal disorders involve tendinopathy, which is characterized by pain, swelling, dysfunction of the tendon and peritendinous tissues, and long-term or permanent deficits in the function of the patient's motor system (2). The current treatment of tendon injuries is mainly non-surgical, mostly aimed at relieving painful symptoms, while the healing treatment of tendon tissue injuries is less effective and often results in weakened tendon function (3). ACL injuries are ligament injuries with a high incidence, with approximately 250,000 ACL injuries occurring each year in the United States (4). ACL injuries often occur during landing, sharp stops, and changing directions (5). The current treatment is mainly surgical and aims to restore structural function, while it takes a long time for the athlete to return to the field. In recent years, the role of platelet-rich plasma(PRP) in muscle, tendon, and ligament healing has become more familiar, especially for the treatment of refractory tendon and ligament degenerative lesions with good clinical efficacy (6–8). Currently, there is literature measuring trends in the use of platelet-rich plasma in osteoarthritis of the knee (9) and orthopedics (10). As platelet-rich plasma continues to be intensively studied in the field of sports medicine and rehabilitation medicine, it is important to explore the research hotspots and trends regarding platelet-rich plasma in the treatment of tendon ligament injuries, but there are currently no relevant bibliometric studies addressing this research area. Therefore, this paper is the first to use a visual research tool to map the scientific knowledge of platelet-rich plasma for tendon ligament injury research. This study only included English-language literature studies from the core dataset of the Web of Science database and may have ignored high-quality literature from other databases or other languages in the field, which has limitations in literature retrieval. Due to the different algorithms, there is no standardized setting process for time partitioning, thresholding, and cropping methods in the process of generating visual atlases for the time being, which may cause some bias. The purpose of this paper is to analyze its hotspots, frontiers and evolutionary paths to gain insight into the research status and development trend of this field, with a view to providing reference and reference for researchers.

The VOSviewer program, created by Professors Van Eck and Waltman, and the CiteSpace software, created by Professor Chen C, co-citation networks are generated using reference citations, revealing the structure of specific research fields. Visualization of critical most-cited documents, research topics, and areas of expertise to detect and visualize trends and patterns within knowledge areas (11). Using CiteSpace 6.1, VOSviewer 1.6.18, and R-bibliometrix 4.6.1 visualization software, the relevant literature in the Web of Science database for the last 20 years was studied in terms of high-impact countries or regions, institutions, authors, journals, keywords, and co-cited literature. We analyzed and tracked the hot spots and cutting-edge trends of using platelet-rich plasma to treat ligament and tendon injuries research and provided some reference and support for the development of research and related disciplines in this field.

## 2. Methods

### 2.1. Methodology data sources and retrieval strategies

**Figure 1:**
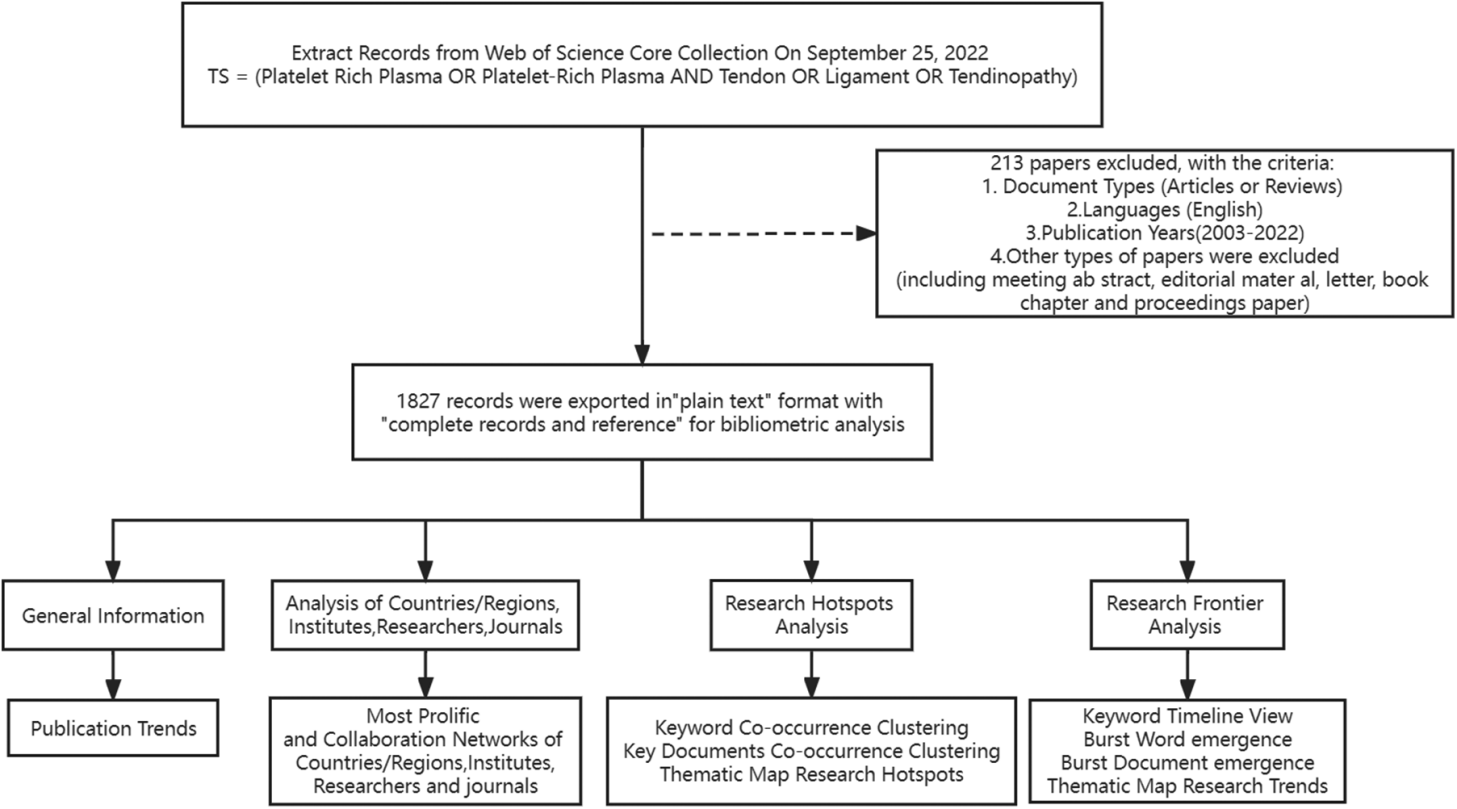
Workflow diagram.

The Web of Science database core dataset was searched.The search results show that the earliest research in this field appeared in 2003, so the search period starts from 2003, the articles were well-representative and peer-reviewed journal papers. Add search terms to the basic search, select “Article” and “Review” for the document type, “English” for the language, and the search period was selected from 2003 to 01–01 to 2022–09–25. The search formula “TS = (Platelet Rich Plasma OR Platelet-Rich Plasma AND Tendon OR Ligament OR Tendinopathy)” was used in the advanced search section to retrieve a total of 2040 relevant articles. A total of 1,827 documents were included after filtering and excluding conference abstracts, proofreading notices, news, conference papers, and retraction notices by the document types included in the Web of Science database. The retrieved literature records were exported in “plain text” format, including “complete records and references”, and a file was generated for every 500 records, renamed as “download”, and then imported into the “input” folder of citespace software, and finally start the visual analysis. The workflow diagram is shown in Figure 1.

### 2.2. Literature selection

Two reviewers independently read the literature. Articles were initially screened by title and abstract before inclusion and exclusion criteria were applied. During screening, if a dispute arises, a third reviewer is assigned to read the entire paper and make a decision.

### 2.3. Data analysis and descriptive analysis

CiteSpace 6.1 identifies the information relationship in literature based on relevant information in articles. According to the parameters setting for CiteSpace 6.1 visualization software, 2003–2022 is the period, one year is the time division, the threshold item is selected as “Top N” and set to 50, then select “Pathfinder” as a cut link to simplify the network structure and highlight key features. For the node type, country (region), institution, author, journal, cited author, keyword, and cited literature are selected for co-occurrence analysis and visualization mapping (12). The visualization was done. We also use CiteSpace to explore the rate of change of keywords and key literature proliferation by using the CiteSpace prominence detection function and generate the ranking table of keywords and key literature with high-intensity mutation rates to analyze the current situation and predict prospects (13). The keyword and key literature ranking table are generated to analyze the current status and predict prospects.

In this paper, three scientometric software and Microsoft Excel programs were used to perform the bibliometric analysis: CiteSpace version 6.1R3, VOSviewer version 1.6.18, and the R-Studio-based visualization software R-bibliometrix version 4.6.1. Citespace and VOSviewer were mainly used to visualize and analyze the knowledge structure and evolutionary trends of the scientific literature in a given field (14). In addition, sub-clusters can be extracted from the overall structure of the literature network through cluster analysis to identify research sub-domains, i.e., research hotspots (15). Identification of research frontiers through overlap analysis using R-bibliometrix, VOSviewer, and CiteSpace software can largely predict the research directions that may yield major breakthroughs in the coming years. Through an in-depth review of the results, researchers can quickly grasp the knowledge base and trends in the field to improve their research results and adjust their research strategies.

## 3. Results

### 3.1. Bibliometrics of publication output

**Figure 2:**
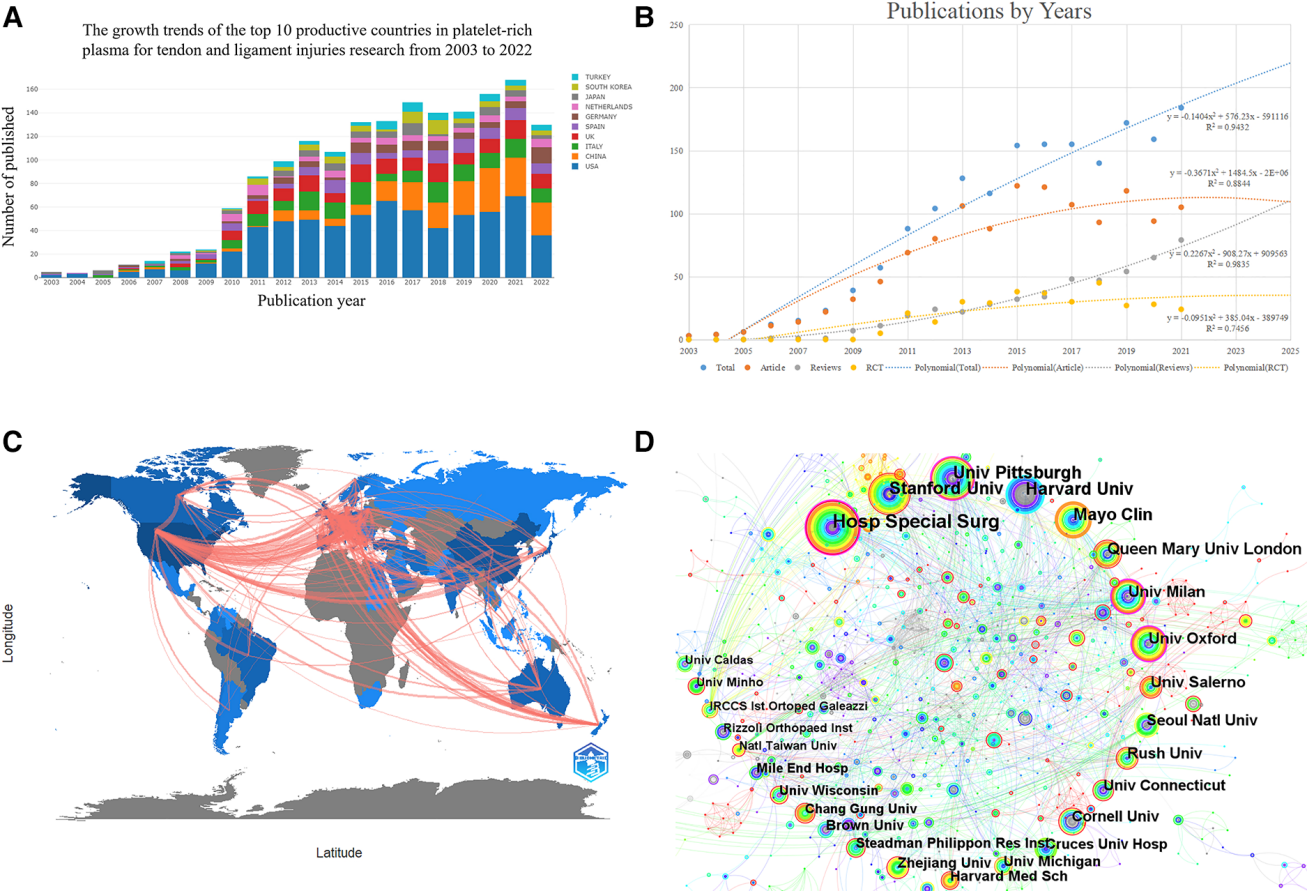
(**A**) bibliometric analysis of the WoS core database output. The number of publications on platelet-rich plasma studies for tendon and ligament injuries published in different countries has changed year by year from 2003 to 2022. (**B**) Publication trends in the field of platelet-rich plasma for tendon and ligament injury research and corresponding polynomial fit curves. (**C**) Collaborative bibliometric analysis of countries in the field of platelet-rich plasma for tendon and ligament injury research, with darker colors representing more publications and connecting lines representing collaborative relationships. (**D**) Institutional co-occurrence network diagram of using platelet-rich plasma to treat ligament and tendon injuries. Note: The circle in the chart indicates the volume of articles issued. The larger the circle indicates the more articles issued by the institution, the thickness of the purple outer circle represents the centrality of the institution, and the connecting line represents the existence of cooperation or co-occurrence.

The Web of Science database is one of the largest citation databases in the world, and the accessibility of this study was obtained through the library of the University of Science and Technology of China. A total of 1,827 papers on using platelet-rich plasma to treat ligament and tendon injuries were retrieved, and Figure 2A shows the annual publication volume in different countries. The number of research papers on using platelet-rich plasma to treat ligament and tendon injuries generally maintained a gradually increasing trend from 2003 to 2022, and in 2010 the field began to grow by leaps and bounds, with nearly three times the number of papers published in 2009. The United States accounts for the largest number of publications per year, followed by China, especially in recent years, with a significant increase year by year.

We discovered substantial connections between the number of publications and the year of publication using a polynomial fit analysis (coefficients of determination (R2) of 0.9432, 0.8844, 0.9835, and 0.7456 for total papers, articles, reviews, and randomized controlled trials, respectively). According to the polynomial fit study, we forecast that there will be around 222 published publications in 2025, comprising of 110 articles, 109 reviews, and 30 randomized controlled trials, as shown in Figure 2B. Overall, the boom in this field in orthopedics and sports medicine has led to an increasing depth of research. However, it can be observed that despite the yearly increase in the number of publications, there still needs to be more high-quality RCT studies.

### 3.2. Countries or regions and cooperation networks

A total of 68 countries or regions were included in the obtained visualization, among which 14 countries or regions have ≥ 50 articles and the highest number of articles is United States with 678 articles, the second is China with 187 articles, and the third is Italy with 172 articles. The visualization of research countries suggests that the top 3 nations in terms of centrality are United States (0.27), ENGLAND (0.19), and SWITZERLAND (0.17). The graph demonstrates that these 3 nations have had successful research partnerships with other nations, see Figure 2C, Table 1.

**Table 1:** Top 10 high-impact countries and institutions for platelet-rich plasma for tendon and ligament injury research.

Country	Number of articles issued	Centrality	Institution	Number of articles issued	Centrality
United States	678	0.27	Hosp Special Surg	56	0.13
PEOPLES R CHINA	187	0.07	Stanford Univ	43	0.09
ITALY	172	0.01	Univ Pittsburgh	43	0.2
ENGLAND	155	0.19	Harvard Univ	40	0.05
SPAIN	113	0.08	Mayo Clin	37	0.06
GERMANY	82	0.08	Queen Mary Univ London	31	0.06
JAPAN	71	0.15	Univ Milan	28	0.13
NETHERLANDS	70	0.14	Univ Oxford	27	0.11
SOUTH KOREA	67	0.03	Univ Salerno	26	0.01
TURKEY	65	0.01	Seoul Natl Univ	25	0.02

### 3.3. Research institutions and cooperation networks

Using platelet-rich plasma to treat ligament and tendon injuries is a hot spot for institutional collaborative research. A total of 1,684 nodes, 3,707 connections, and a topological network density of 0.0026 were generated. 1,684 institutions published research on using platelet-rich plasma to treat ligament and tendon injuries, with 14 institutions publishing ≥ 20 articles, the most prolific being Hosp Special Surg (New York Hospital for Special Surgery) with 56 articles, the second being Stanford Univ (Stanford University) with 43 articles, and the third being Univ Pittsburgh (University of Pittsburgh) with 43 articles. Univ Pittsburgh (University of Pittsburgh) with 43 articles; the top 3 institutions in terms of centrality, as suggested by the visualization of research institutions, were Univ Pittsburgh (0.2), Hosp Special Surg (0.13), and Univ Milan (0.13). These three institutions have well-established research collaborations with other institutions. See Figure 2D, Table 1.

### 3.4. High-Impact authors and collaborative networks

**Figure 3:**
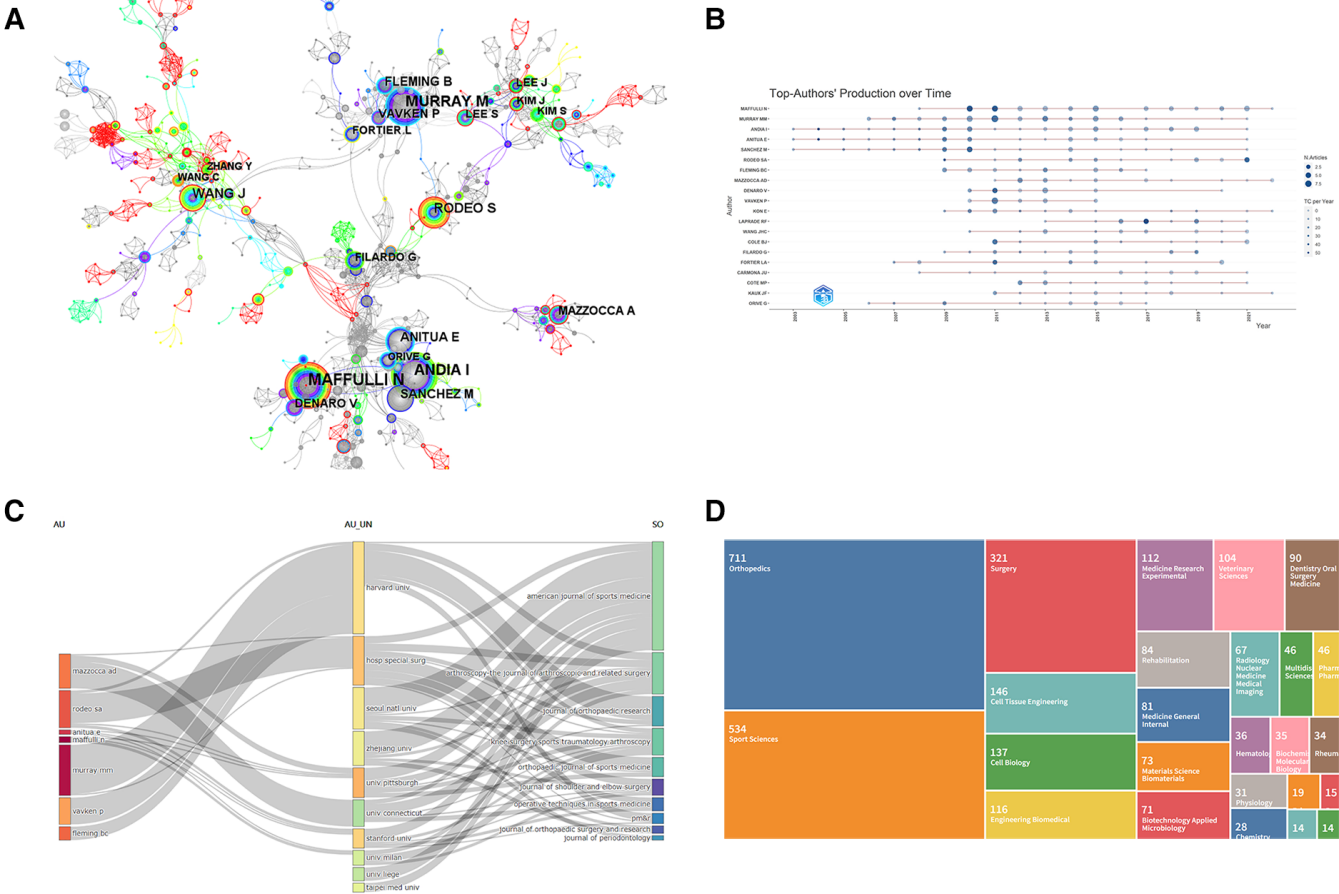
(**A**) author co-occurrence network diagram of using platelet-rich plasma to treat ligament and tendon injuries. Note: The circles in the chart indicate the volume of posts. The larger the circle, the more the author posts, the thickness of the purple outer circle represents the author's centrality, and the connecting line represents the existence of cooperation or co-occurrence. (**B**) Plot of high yield authors over time for using platelet-rich plasma to treat ligament and tendon injuries. Note: The top twenty most prolific researchers in the field and their publications. The larger the node, the more literature is published. The darker the color, the more citations. The color represents the number of publications, and the color represents the number of citations per year. (**C**) The three-field plot shows the knowledge flow of platelet-rich plasma for the treatment of tendon and ligament injuries. (**D**) Distribution of hot research disciplines in the literature on using platelet-rich plasma to treat ligament and tendon injuries.

The most prominent author was Maffulli N, University of Salerno, Italy (48 articles); the second most frequent author was Murray M, Harvard Medical School, United States (42 articles); the third most frequent author was Andia I, University of Cruces, Spain (34 articles); the highest document centrality was achieved by Zhang J, University of Pittsburgh, United States and Su CA, Case Western Reserve University, United States, both with 0.07 centrality. The highest cited authors are Anitua E of the Eduardo Anitua Institute of Biomedical Research, Spain (478 citations), followed by Mishra A of Stanford University, United States (390 citations) and Anton Anton, The Netherlands. (390 times) and de Vos RJ, Antoniushove Medical Center, The Hague, The Netherlands (389 times). Among the highly prolific posting authors, the research team represented by Italian scholars Maffulli N, Denaro V, and Longo UG has a strong collaborative relationship and forms a research group with a dense aggregation. The results of the members of this team regarding this study are focused on clinical studies and experimental animal models of platelet-rich plasma on tendinopathy; the research team centered on Murray M, Vavken *P* and Fleming B from Harvard Medical School, United States, whose research area is collagen-platelet-rich plasma hydrocoagulation scaffold promotion on histological repair of central anterior cruciate ligament wounds The research team is centered on Andia I, Sanchez M and Anitua E from the University of Cruces, Spain, whose members are working on the *in vitro* cellular molecular biology of growth factor (PRGF)-rich preparations on tendon cells, the molecular biology of platelets in healing and tissue regeneration and their potential impact on different disciplines; and the research team is centered on Wang J, Zhang J and Zhang Y of the University of Pittsburgh, United States, the research team whose members' research area is the cellular molecular biology of platelet-rich plasma release agents promoting the differentiation of tendon stem cells into live nature cells. The figure shows that there is some author team collaboration, however it is more pronounced among the high-yield authors and there aren't many authors who are high centrality to the literature, see Figure 3A–C, Table 2.

**Table 2:** Top 10 high-impact authors of platelet-rich plasma studies for tendon and ligament injuries.

Author	Country	Institution	Number of articles issued	Author	Frequency of citations	Country
Maffulli N	Italy	Univ Salerno	48	Anitua E	478	Spain
Murray MM	United States	Harvard Medical School	42	Mishra A	390	United States
Andia I	Spain	Univ Cruces	34	de Vos RJ	389	Netherlands
Rodeo SA	United States	Hosp Special Surg	25	Sánchez M	367	Spain
Wang JH	United States	Univ Pittsburgh	22	Marx RE	355	United States
Anitua E	Spain	Eduardo Anitua Institute of Biomedical Research	20	Maffulli N	277	Italy
Sánchez M	Spain	USP-La Esperanza Clinic	18	Filardo G	273	Italy
Proffen BL	United States	Harvard Medical School	17	Jo CH	246	Korea
Vavken *P*	United States	Harvard Medical School	16	Kon E	237	Italy
Mazzocca AD	United States	Univ Connecticut	15	Peerbooms JC	229	Netherlands

### 3.5. Hot disciplines in literature research

The distribution of research hotspots in the literature was analyzed, and 25 categories were created from the 1,827 documents that were successfully recovered. Among them, 711 were in the orthopedic category, 534 in the sports science category, and 321 in the surgery category. From the data, it can be seen that using platelet-rich plasma to treat ligament and tendon injuries has been extensively researched and developed in orthopedics, sports science, and surgery, Figure 3D.

### 3.6. High impact journals and citation relationships

**Figure 4:**
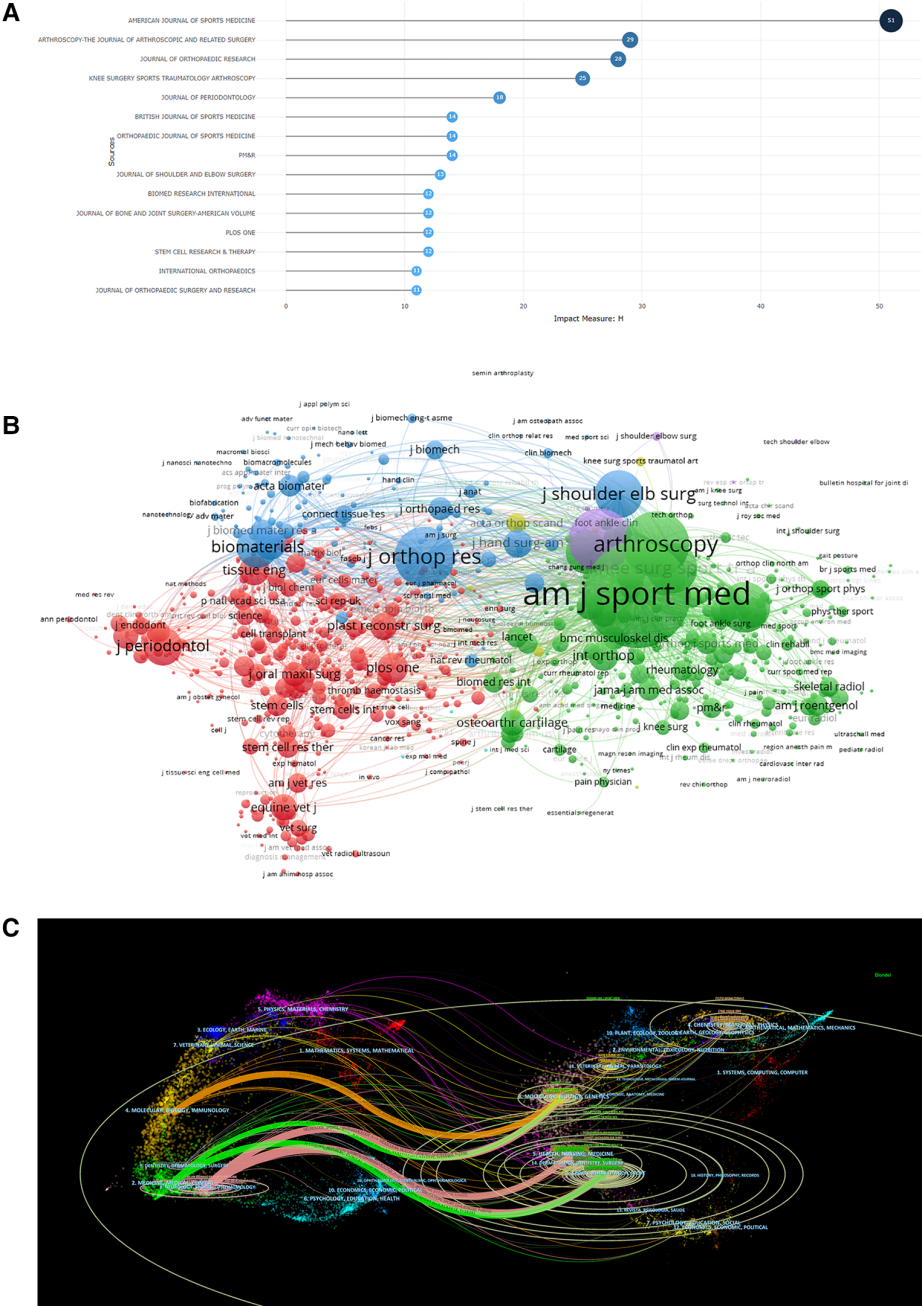
(**A**) H-index of high-impact journals for platelet-rich plasma for tendon and ligament injury research, 2003-2022. (**B**) Cluster visualization of journal co-citation analysis generated based on VOSviewer software. Note: Each node represents a journal, and the size of each circle is determined by the journal's co-citation. (**C**) Double graph overlay of cited and cited journals in the field of platelet rich plasma for tendon and ligament injury research. Note: Cited journals are on the left, cited journals are on the right, and the line represents the citation status.

The top three journals in terms of the number of articles were AMERICAN JOURNAL OF SPORTS MEDICINE (121 articles, total citations 3854, average citations 31.85), ARTHROSCOPY-THE JOURNAL OF ARTHROSCOPIC AND RELATED SURGERY (71 articles, total citations 1119, average citations 15.76) and KNEE SURGERY SPORTS TRAUMATOLOGY ARTHROSCOPY (53 articles, total citations 484, average citations 9.13). (53 citations, 484 total citations, 9.13 average citations). The top three journals with high H-index for using platelet-rich plasma to treat ligament and tendon injuries were AMERICAN JOURNAL OF SPORTS MEDICINE (16), ARTHROSCOPY-THE JOURNAL OF ARTHROSCOPIC AND RELATED SURGERY (17), and JOURNAL OF ORTHOPAEDIC RESEARCH (18); based on the co-citation analysis of journals produced by VOSviewer, platelet-rich plasma The top three cited research studies for the treatment of tendon and ligament injuries were AMERICAN JOURNAL OF SPORTS MEDICINE (1090), JOURNAL OF BONE AND JOINT SURGERY-AMERICAN VOLUME (849), and JOURNAL OF ORTHOPAEDIC RESEARCH (770) Figure 4A,B, Table 3. currently, the impact of journals publishing in this research area is high.

**Table 3:** Status of high-impact journals for platelet-rich plasma for tendon and ligament injury research, 2003-2020.

Journal Name	Total number of articles	Total number of applications	The average number of citations	IF (2022)	JCR (2022)	H-index
AMERICAN JOURNAL OF SPORTS MEDICINE	121	3854	31.85	7.01	Q1	51
ARTHROSCOPY-THE JOURNAL OF ARTHROSCOPIC AND RELATED SURGERY	71	1119	15.76	5.97	Q1	29
KNEE SURGERY SPORTS TRAUMATOLOGY ARTHROSCOPY	53	484	9.13	4.11	Q1	25
ORTHOPAEDIC JOURNAL OF SPORTS MEDICINE	49	159	3.24	3.40	Q2	14
JOURNAL OF ORTHOPAEDIC RESEARCH	46	1235	26.85	3.1	Q1	28
JOURNAL OF PERIODONTOLOGY	29	278	9.59	4.49	Q1	18
PM & R	29	243	8.38	2.29	Q3	14
OPERATIVE TECHNIQUES IN SPORTS MEDICINE	24	42	1.75	0.31	Q4	7
JOURNAL OF ORTHOPAEDIC SURGERY AND RESEARCH	23	80	3.48	2.67	Q3	11
JOURNAL OF SHOULDER AND ELBOW SURGERY	22	321	14.59	3.50	Q2	13

The biplot overlay's colored paths connecting journal groups depict the citation relationships between the citing and cited journals, illuminating the knowledge flow and citation trajectory (19). The colored paths indicate that studies published in pharmaceutical/medical/clinical journals typically cite studies published in sports/rehabilitation/exercise, health/nursing/medicine, molecular/biology/genetics, and veterinary/zoology/quarantine.More information about representative citations and cited journals in each cluster can be found in Figure 4C. For instance, the American Journal of Sports Medicine, Arthroscopy, Journal of Bone and Joint Surgery American Volume, Journal of Orthoptera Research, and British Journal of Sports Medicine are the most typical journals in the Sports/Rehab/Exercise cluster.

## 4. Keyword visualization analysis

### 4.1. Analysis of research hotspots based on keyword co-occurrence clustering

**Figure 5:**
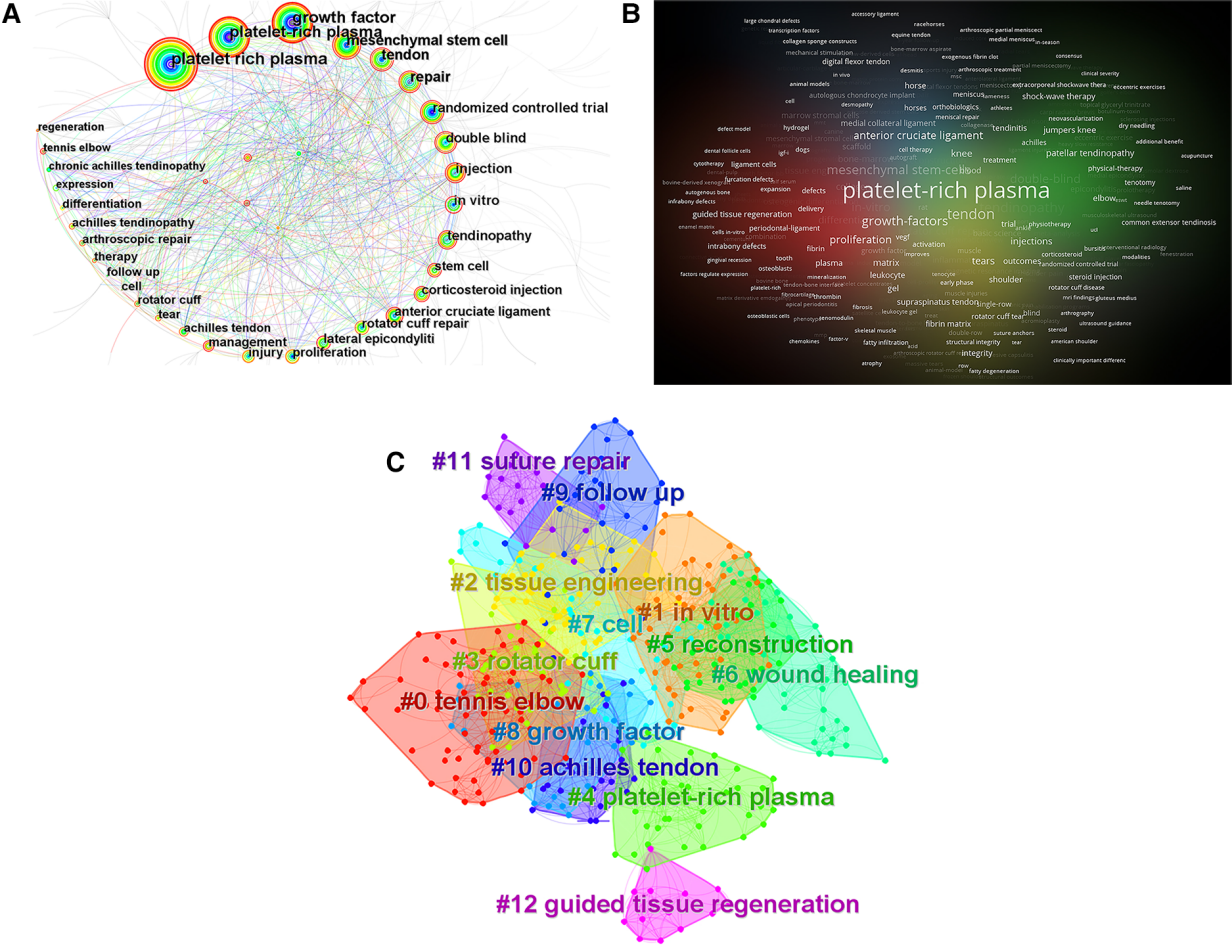
(**A**) keyword co-occurrence plot of using platelet-rich plasma to treat ligament and tendon injuries. Note: The circles in the figure represent keywords, and the larger the circle indicates the higher the frequency of the keyword. The dark to light colors represent the years from far to near, the connecting lines represent the links between the keywords, and the thickness of the purple outer circle represents the centrality of the keywords. (**B**) VOSviewer-based clustering density view of using platelet-rich plasma to treat ligament and tendon injuries keywords. (**C**) Cooccurrence clustering of keywords for using platelet-rich plasma to treat ligament and tendon injuries. Note: Dark to light colors represent years from distant to recent, and connecting lines represent associations between keywords.

Keyword analysis plays a key role in summarizing research hotspots and exploring research trends (14). After removing the subject terms related to the search strategy, a topological network density of 0.0094 was developed, resulting in a total of 481 nodes and 1,084 connections, and the keywords with higher co-occurrence are represented in Figure 5A,B, Table 4. The research hotspots at that time are represented by keywords with a higher centrality and frequency. Analysis of popular keywords revealed that research in this field focused on growth factor, mesenchymal stem cell, repair, randomized controlled trial, double blind, injection, *in vitro*, stem cell, corticosteroid injection, anterior cruciate ligament, rotator cuff repair, proliferation, lateral epicondylitis, injury, management, achilles tendon, and follow up. Figure 5C exhibits the keyword co-occurrence clustering map in this domain. Using the classical LLR algorithm, a total of 13 clusters were formed, and the keyword clustering analysis showed that the homogeneity between research was greater the higher the degree of aggregation (20). The higher the degree of aggregation, the better the homogeneity among studies. The cluster number is inversely proportional to the cluster size, with the largest cluster marked by #0 and so on. The keyword clusters from #0 to #12 were tennis elbow, *in vitro*, tissue engineering, rotator cuff, platelet-rich plasma, reconstruction, wound healing, cell, growth factor, follow up, achilles tendon, suture repair, and guided tissue regeneration. The clusters #0, #2, #3, #7, #8, #9, and #10 are most intersectingly linked, followed by #2, #9, and #11 intersectingly linked, and #4, #12 intersectingly linked. Keyword co-occurrence and cluster analysis yielded that tennis elbow, *in vitro*, anterior cruciate ligament, rotator cuff repair, mesenchymal stem cell, growth factor, follow up, achilles tendon, suture repair, and guided tissue regeneration are current research hotspots in this field.

**Table 4:** Using platelet-rich plasma to treat ligament and tendon injuries high frequency keywords and centrality TOP10.

Keywords	Frequency	Keywords	Centrality
Growth factor	510	Repair	0.26
Mesenchymal stem cell	301	Wound healing	0.22
Repair	246	Growth factor	0.12
Randomized controlled trial	239	*P*eriodontal ligament	0.11
Double-blind	233	Reconstruction	0.11
Injection	202	tgf beta 1	0.1
In vitro	191	Transplantation	0.1
Stem cell	167	Therapy	0.09
Corticosteroid injection	162	Injury	0.09
Anterior cruciate ligament	158	Cell	0.09

### 4.2. Trend analysis of research frontiers based on the keyword timeline view

**Figure 6:**
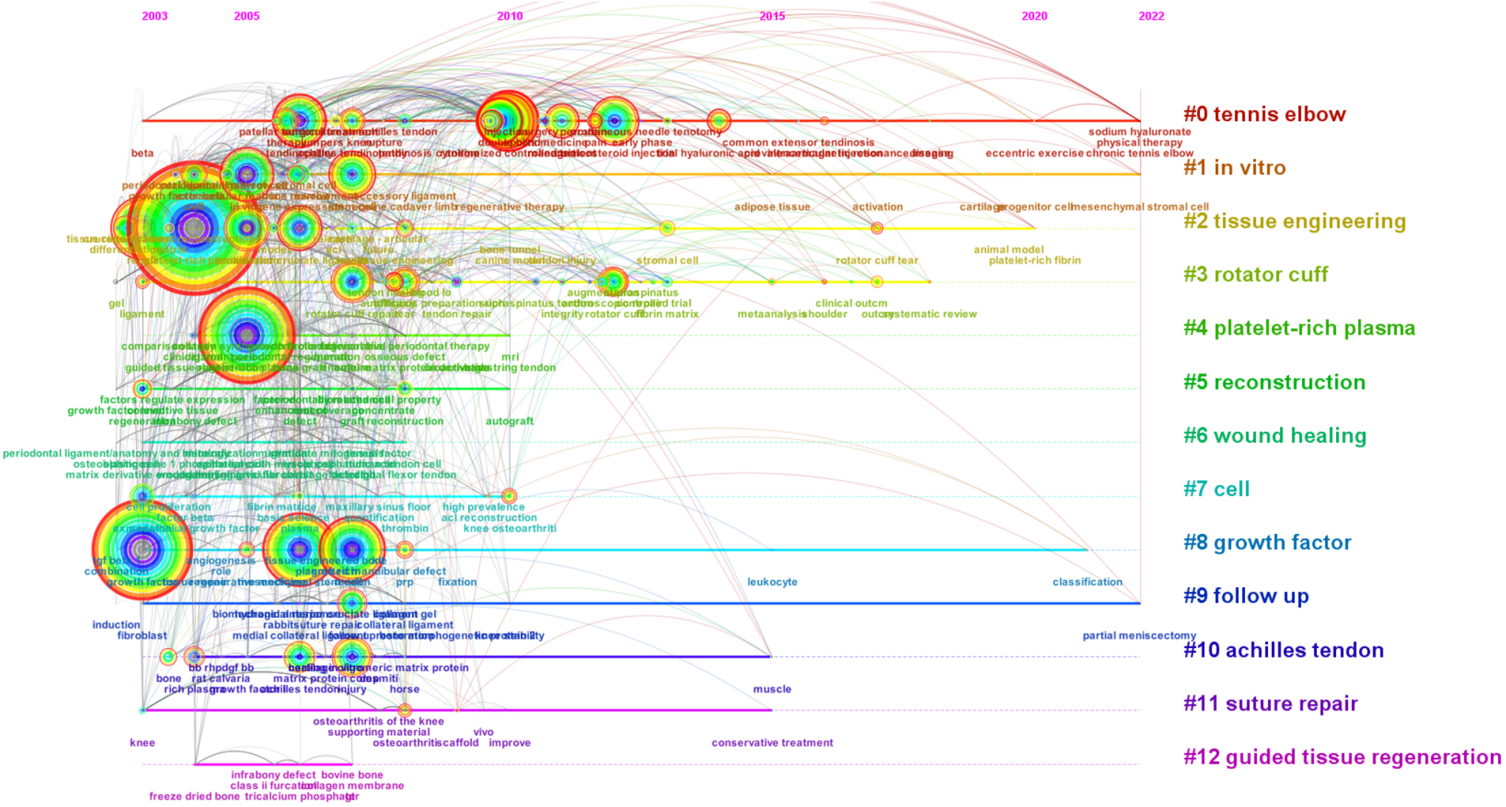
Keyword timeline view of using platelet-rich plasma to treat ligament and tendon injuries. Note: The circles in the figure represent keywords, and the larger the circle indicates the higher the frequency of the keyword. The dark to light colors represent the years from far to near, the connecting lines represent the links between the keywords, and the thickness of the purple outer circle represents the centrality of the keywords.

A timeline view of the literature that the Web of Science database has filtered reveals the temporal dynamics of the clustered keywords, Figure 6. Cluster modularity (Q value) = 0.6433 > 0.5, indicating significant cluster structure; cluster Silhouette (S value) = 0.8587 > 0.5, indicating convincing clustering (21). From 2003 to 2005, the keywords repair, expression, growth factor, differentiation, extracellular matrix, cell, platelet-rich plasma, ligament, *in vitro*, matrix, cruciate ligament reconstruction, plasma-rich, endothelial growth factor, gel, growth factor beta, guided tissue regeneration, proliferation and regenerative medicine received extensive attention; from 2005 to 2010, the keywords treatment, tendinopathy, achilles tendinopathy, gene expression, stem cells, anterior cruciate ligament, tissue engineering, rotator cuff repair, tear, efficacy, tendon repair, surgical treatment, sports medicine, jumpers knee, patellar tendon, *in vivo*, patellar tendinopathy, reconstruction, graft, mesenchymal stem cells, tendon, platelet-rich plasma, follow up, achilles tendon and injury began to receive attention; 2010 to 2015 chronic achilles tendinopathy, injection, lateral epicondylitis, double blind, tennis elbow, randomized controlled trial, management, corticosteroid injection, hyaluronic acid, pain, stromal cells, fibrin matrix, arthroscopic repair, rotator cuff and anterior cruciate ligament reconstruction are getting attention; 2015 to 2020 intra-articular injection, rotator cuff tear, clinical outcome, meta-analysis, conservative treatment and muscle are getting attention; 2020 to 2022 centrifugal motion, progenitor cells, sodium hyaluronate, platelet-rich fibrin, physical therapy, greater trochanteric pain syndrome, classification, and partial meniscectomy are new terms. It is predicted that using platelet-rich plasma to treat ligament and tendon injuries will continue to be studied in depth around centrifugal exercise therapy, sodium hyaluronate, physical therapy, platelet-rich fibrin for external epicondylitis, ex vivo injection of progenitor cells and mesenchymal stem cells for rotator cuff tears, and classification and follow up related to growth factors.

### 4.3. Trend analysis of research frontiers based on emergent word emergence

**Figure 7:**
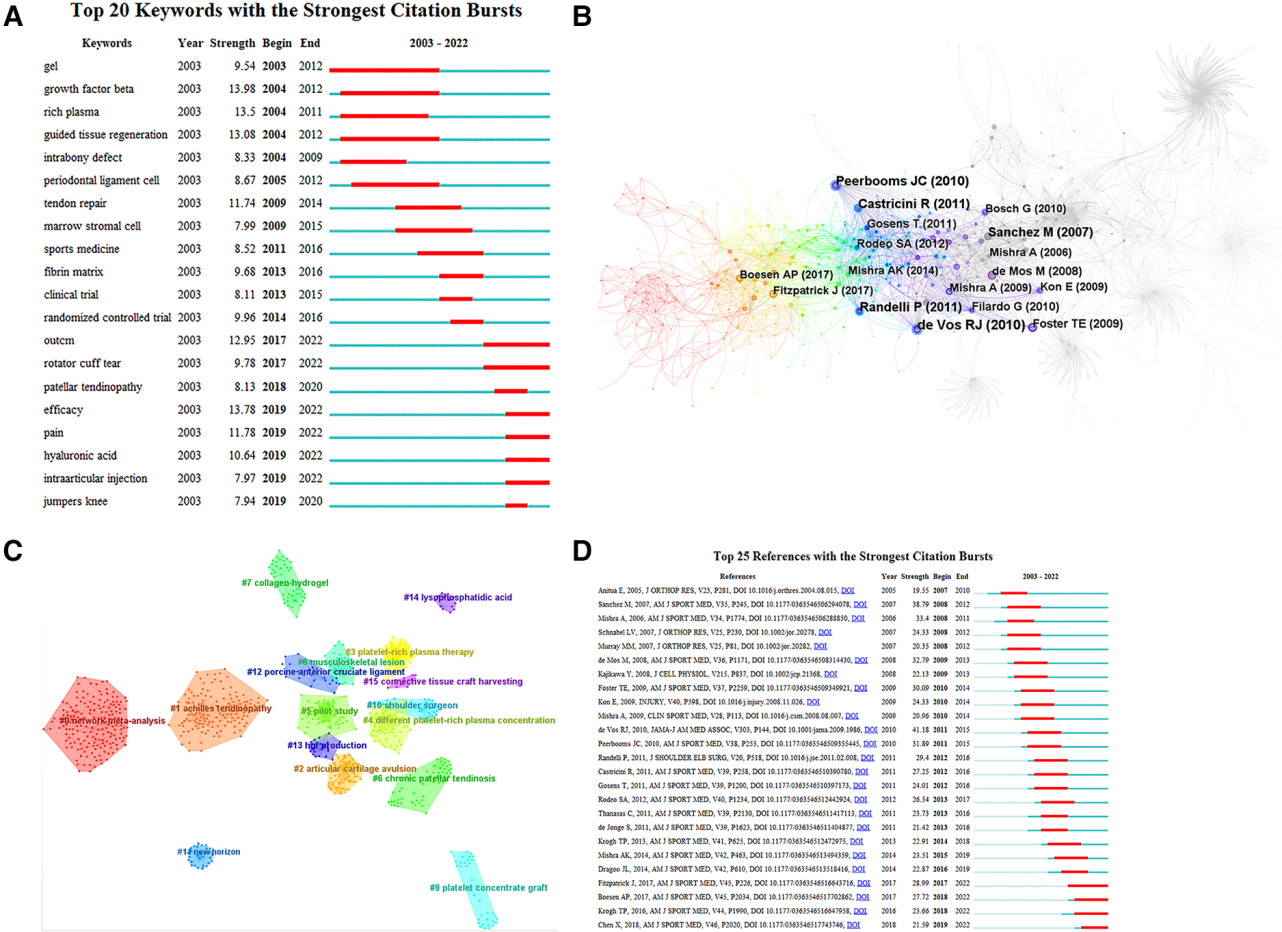
(**A**) keyword emergent plots of using platelet-rich plasma to treat ligament and tendon injuries. Note: The figure of “–” is 1 year keyword mark, “–” is the emergence of the year of the word. (**B**) Co-citation network of using platelet-rich plasma to treat ligament and tendon injuries. Note: The circles in the figure represent keywords, the larger the circle means the higher the frequency of keywords, the dark to light color represents the year from far to near, the connecting line represents the connection between keywords, the thickness of the purple outer circle represents the centrality of keywords, this figure is arranged in chronological order from dark to light by clustering color. (**C**) Clustering of co-cited literature on using platelet-rich plasma to treat ligament and tendon injuries. Note: The circles in the figure represent keywords, the larger the circle means the higher the frequency of keywords, the dark to light color represents the year from far to near, the connecting line represents the connection between keywords, the thickness of the purple outer circle represents the centrality of keywords, this figure is arranged in chronological order from dark to light by clustering color. (**D**) Summary of key literature on using platelet-rich plasma to treat ligament and tendon injuries. Note: The “–” in the figure are the years in which the unexpected citations appeared, and the “–” in the figure are the nodes in which the number of citations for the unexpected citations suddenly rose, arranged in chronological order from top to bottom.

Among the 20 mutation words with the highest frequency of cited literature screened by the Web of Science database, mutation words represent keywords that are frequently cited during a certain period of time, such that hotspots and trends can be identified. There are 8 mutation words with a mutation period from 2017 to 2022. The strongest mutation was “growth factor beta” (13.98), followed by “efficacy” (13.78), and in third place was “guided tissue regeneration” (13.08). The keywords with high mutation intensity in the past 5 years include: “outcm” (2017–2022), “rotator cuff tear” (2017–2022), patellar tendinopathy” (2018–2020). Figure 7A. Combining the temporal dynamics of the keywords and Burst analysis, it is possible to form a general picture of the development and future research trends in the field of using platelet-rich plasma to treat ligament and tendon injuries. “outcm”, “rotator cuff tear”, “efficacy”, “pain”, “ hyaluronic acid” and “intraarticular injection” all continue to be studied to this day and are likely to remain so.

## 5. Visual analysis of key documents

### 5.1. Analysis of research hotspots based on key literature

A total of 1,827 papers were retrieved in this field, with a total citation frequency of 8,136, and the co-citation graph of research literature generated 1,131 nodes and 3,594 connections, with a topological network density of 0.0056. The top 10 rankings of literature with high citation frequency and high centrality are shown in Figure 7B, Tables 5, 6. One of the key markers of the research hotspots is the citation situation and centrality, and by studying the literature that has a lot of citations and centrality. It is possible to comprehend some research findings carefully in order to identify the areas of interest in the field. The top 10 documents with high citation and centrality were read and analyzed.

**Table 5:** Top 10 cited literature centrality rankings for using platelet-rich plasma to treat ligament and tendon injuries.

Author	Centrality	Year	Title	Periodicals
de Vos RJ (22)	0.35	2010	Platelet-rich plasma injection for chronic achilles tendinopathy: a randomized controlled trial	JAMA-J AM MED ASSOC
Sanchez M (23)	0.3	2007	Comparison of surgically repaired achilles tendon tears using platelet-rich fibrin matrices	AM J SPORT MED
de Vos RJ (24)	0.21	2014	Strong evidence against platelet-rich plasma injections for chronic lateral epicondylar tendinopathy: a systematic review	BRIT J SPORT MED
Murray MM (25)	0.18	2007	Enhanced histologic repair in a central wound in the anterior cruciate ligament with a collagen-platelet-rich plasma scaffold	J ORTHOP RES
Molloy T (26)	0.16	2003	The roles of growth factors in tendon and ligament healing	SPORTS MED
Krogh TP (27)	0.12	2013	Treatment of lateral epicondylitis with platelet-rich plasma, glucocorticoid, or saline: a randomized, double-blind, placebo-controlled trial	AM J SPORT MED
Anitua E (28)	0.12	2003	Autologous platelets as a source of proteins for healing and tissue regeneration	INT J ORAL MAX IMPL
Fitzpatrick J (18)	0.11	2017	The Effectiveness of Platelet-Rich Plasma in the Treatment of Tendinopathy: A Meta-analysis of Randomized Controlled Clinical Trials	AM J SPORT MED
Anitua E (17)	0.11	2006	Autologous fibrin matrices: a potential source of biological mediators that modulate tendon cell activities	J BIOMED MATER RES A
Marx RE (29)	0.11	2004	Platelet-rich plasma: evidence to support its use	J ORAL MAXIL SURG

**Table 6:** Top 10 cited literature frequency ranking of using platelet-rich plasma to treat ligament and tendon injuries.

Author	Frequency of citations	Year	Title	Periodicals
de Vos RJ (22)	158	2010	Platelet-rich plasma injection for chronic achilles tendinopathy: a randomized controlled trial	JAMA-J AM MED ASSOC
Peerbooms JC (30)	119	2010	Positive effect of an autologous platelet concentrate in lateral epicondylitis in a double-blind randomized controlled trial: platelet-rich plasma versus corticosteroid injection with a 1-year follow up	AM J SPORT MED
Castricini R (31)	105	2011	Platelet-rich plasma augmentation for arthroscopic rotator cuff repair: a randomized controlled trial	AM J SPORT MED
Randelli *P* (32)	94	2011	Platelet rich plasma in arthroscopic rotator cuff repair: a prospective RCT study, 2-year follow up	J SHOULDER ELB SURG
Sanchez M (23)	91	2007	Comparison of surgically repaired achilles tendon tears using platelet-rich fibrin matrices	AM J SPORT MED
Foster TE (6)	90	2009	Platelet-rich plasma: from basic science to clinical applications	AM J SPORT MED
de Mos M (33)	87	2008	Can platelet-rich plasma enhance tendon repair? A cell culture study	AM J SPORT MED
Rodeo SA (34)	78	2012	The effect of platelet-rich fibrin matrix on rotator cuff tendon healing: a prospective, randomized clinical study	AM J SPORT MED
Gosens T (35)	77	2011	Ongoing positive effect of platelet-rich plasma versus corticosteroid injection in lateral epicondylitis: a double-blind randomized controlled trial with 2-year follow up	AM J SPORT MED
Filardo G (36)	74	2010	Use of platelet-rich plasma for the treatment of refractory jumper's knee	INT ORTHOP

The key node literature is usually the literature that presents significant theoretical or innovative concepts in the field and is also the literature that is most likely to generate new research hotspots. Based on the analysis of the co-citations of the key nodes and the most frequently cited literature, we can sort out the research hotspots and evolutionary trajectory of platelet-rich plasma in the field of tendon ligament injury treatment. These 20 pieces of literature are the most important classical literature in the study and can represent the key research contents in the field. Based on the classification of research objects, they can be divided into 3 categories: clinical studies, *in vitro* experimental studies and reviews. The 1st, 2nd, 6th centrality and 1st, 2nd, 3rd, 4th, 5th, 8th, 9th, and 10th citation frequency are clinical studies, the 4th, 9th centrality and 7th citation frequency are *in vitro* experimental studies, and the 3rd, 5th, 7th, 8th, 10th centrality and 6th citation frequency are review studies. The highly cited literature in clinical studies involved achilles tendinopathy (centrality 1, 2 and cited frequency 1, 5 studies), humeral epicondylitis (centrality 3, 6 and cited frequency 2, 9 studies), rotator cuff tears (cited frequency 3, 4, 8 studies), patellar tendinitis (cited frequency 10 studies) and anterior cruciate ligament tears (centrality 4 studies), respectively. According to research, tennis elbow, rotator cuff tendon damage, achilles tendon tendinopathy, and patellar tendinopathy had the highest prevalence of various tendinopathies (37). The results of the study were consistent with the highly cited literature. The research methods used in the literature were double-blind, randomized controlled method (centrality 6th and cited frequency 2nd and 9th studies) and experimental method (centrality 1st, 2nd and cited frequency 1st, 3rd, 4th, 5th, 8th and 10th studies), and for the assessment of treatment outcomes, most of them used visual analog rating scale as a test indicator, as shown in Table 7 which examines the clinical treatment of tendon ligament injuries in the above mentioned 20 significant literature The additional bioactive compounds, doses, therapies and clinical outcomes of the studies are summarized. The effectiveness of platelet-rich plasma in the treatment of tendon ligament injuries was illustrated by comparing the changes in the values of the visual analog scale through a certain period of follow up. By using platelet-rich plasma for the treatment of different tendinopathies, the results of all clinical studies showed that the use of platelet-rich plasma and its related agents had a better effect on tendon and ligament injuries in terms of long-term prognosis, with significant differences in visual analog score scale values before and after treatment. However, in the study with the 1st literature centrality and 1st citation frequency, the authors found that although there was a significant change in the visual analog score values during the follow up period after the use of platelet-rich plasma, no significant difference was observed when compared with other controls.

**Table 7:** Additional bioactive compounds, dose, therapy, quantity, duration and clinical outcomes of PRP in the adjunctive treatment of tendon ligament healing.

Part	Acute or chronic injury	Bioactive compounds	Dosage	Treatment/control	Quantity	Time	Clinical outcomes (indicators)
Achilles tendon	Chronic achilles Tendon Tear (22)	No activator	5 ml PRP	GPS/Saline	54	24 weeks	Both groups improved pain scores and activity levels (VISA-A)
	Open suture complete tear of achilles tendon (23)	Calcium chloride	4 ml PRGF	PRGF II/RIR	12	12 months	Faster return to normal mobility, gentle running and normal training
External epicondyle	Chronic epicondylitis (27)	No activator	3-3.5 ml PRP	GPS/CSI/saline	60/17^a^	12 months	CSI has a short-term pain-reducing effect (PRTEE)
	Chronic epicondylitis (30)	No activator	3 ml PRP	GPS/CSI	100	December	Progressive Pain Relief (DASH)
	Chronic epicondylitis (35)	No activator	Not mentioned	LR-PRP/CSI	100	24 months	Reduces pain and significantly enhances DASH function
Shoulder Sleeve	Repair of rotator cuff tears with small to medium-sized double-row anchor sutures (31)	Calcium chloride	One layer of flat circular PRFM film	PRFM/none	88	16 months	Failure to improve healing of small to medium rotator cuff tears repaired with double-row anchor staples (Constant-Murley)
	Repair of complete rotator cuff tears with single-row anchor staple closure (32)	Autologous thrombin component serum	6 ml PRP	GPS/none	53	24 months	Short-term postoperative pain relief and facilitation of rotator cuff external rotation strength recovery in subgroups with grade 1 and 2 tears (SST)
	Repair of rotator cuff tears with small to medium-sized double-row anchor sutures (34)	Calcium chloride	One layer of flat circular PRFM film	PRFM/none	79	12 weeks	Transosseous equivalent double-row restorations treated with PRFM are more likely to fail
Patellar Tendon	Chronic refractory patellar tendinopathy (36)	Calcium chloride	5 ml PRP three times every two weeks	PRP/none	15	6 months	Significant improvement in both pain scores and functional recovery (Tegner, EQ VAS)
Ligaments	Complete tear of the anterior cruciate ligament (25)	Acid-soluble type I collagen	One type I collagen sponge and 700 ml of hydrogel mixture	Collagen-PRP Hydrogel/none	17^b^	6 months	Improved histological differences in non-healing intra-articular ligament wounds and enhanced expression of related proteins

PRP, PRP without specific preparation device specified; GPS, GPS kit; PRGF II, PRGF System II; LR-PRP, leukocyte-rich platelet plasma, no kit specified; Collagen-PRP Hydrogel, type I collagen sponge strip and hydrogel mixture; CSI, corticosteroid injection; Saline, saline injections; None, same physical therapy or training; RIR, retrospective comparative study; PRTEE, patient-rated tennis elbow; Constant-Murley, Scoring System Shoulder Scale; SST, Simple Shoulder Test; DASH, disability of the arm, shoulder, and hand; Tegner, knee motion score. ^a^indicates only 17 patients remaining by December. ^b^indicates the right and left knees of 17 dogs.

Notably, in the 2nd most cited study, the investigators compared autologous platelet-rich plasma with corticosteroid injections for the first time as a treatment option for patients with failed nonsurgical treatment of humeral epicondylitis to investigate the efficacy of platelet-rich plasma vs. corticosteroids in the treatment of chronic epicondylitis. Prior to the publication of this study, corticosteroid injections for tendinopathy had been used as the gold standard for the treatment of epicondylitis, and while this treatment had good short-term efficacy, adverse outcomes were often seen after 12 weeks of follow up (38). A single injection of platelet-rich plasma provided better improvements in pain and dysfunction than corticosteroid injections, and these improvements persisted over time. In addition, in the Cited Frequency 10 study, investigators used multiple injections of platelet-rich plasma as a treatment modality for patients with chronic jumper's knee disease who failed both nonsurgical or surgical treatment, exploring the efficacy of multiple consecutive platelet-rich plasma injections for chronic jumper's knee disease, after the vast majority of previous studies were single injections with little or late onset of treatment effects for chronic tendinopathy (22, 27). Successive PRP injections have shown satisfactory clinical results in difficult cases of chronic refractory tendinopathy after the failure of previous classical treatments. In all clinical trial studies involving rotator cuff tear repair, investigators have used platelet-rich fibrin matrix (PRFM) as an adjunct to repair. Autologous PRFM does not improve the healing of small to medium rotator cuff tears repaired with double-row anchor staples and may even have a negative effect. PRFM promotes pain reduction and recovery of external rotation strength in patients with complete rotator cuff tears repaired with single-row anchor staples.

In an *in vitro* experimental study, three papers selected complete tears of the anterior cruciate ligament in dogs (centrality study 4) and human tendon cells (centrality study 9 and cited frequency study 7) as subjects respectively. Repaired complete tears of the anterior cruciate ligament in dogs using collagen-PRP stent links, and measured fibronectin, fibrinogen, PDGF-A, TGF-b1, FGF-2, procollagen I, and vWF (revascularization markers) levels were measured in the ACL after a period of repair and compared with the repair of complete tears of the medial collateral ligament, with the aim of investigating whether collagen-PRP hydrogel could partially improve the repair as a temporary replacement scaffold at the ACL wound site. Platelet-rich plasma (centrality study #9), platelet-rich plasma clot releasers, and platelet-poor plasma clot releasers (cited frequency study #7) at different concentrations were selected as culture media for tendon or tendon cells. After a period of culture, the expression of type I collagen, type III collagen, cartilage oligomeric matrix proteins, matrix metalloproteinases, and the expression of proteins or protein genes related to tendon cell anabolism, such as vascular endothelial growth factor, hepatocyte growth factor, and transforming growth factor, were measured in tendon or tendon cells, with the aim of studying the cellular, molecular biology related to platelet-rich plasma on tendon cell synthesis.

### 5.2. Analysis of research hotspots based on co-occurrence clustering of key literature

Cluster analysis based on co-cited literature can present subfields that represent major research hotspots (39). Cluster modularity (Q value) = 0.8231 > 0.5, indicating significant cluster structure; cluster silhouette (S value) = 0.8681 > 0.5, indicating convincing clustering (21). The classical LLR algorithm was used. The classical LLR algorithm was used to form a total of 16 clusters, and the keyword clustering analysis showed that the homogeneity between research was greater the higher the degree of aggregation (20). The cluster number is inversely proportional to the cluster size, and the largest cluster is marked with #0, and so on, Figure 7C. The keyword clusters from #0 to #15 are network meta-analysis, achilles tendinopathy, articular cartilage avulsion, platelet-rich plasma therapy, different platelet-rich plasma concentration, pilot study, chronic patellar tendinosis, collagen hydrogel, musculoskeletal lesion, platelet concentrate graft, shoulder surgeon, new horizons, porcine anterior cruciate ligament, hgf production, lysophosphatidic acid, and connective tissue craft harvesting. In terms of the order of clustering, the top research trends in the field were literature review of network meta-analysis, achilles tendinopathy, different platelet-rich plasma concentrations, pilot studies, and chronic patellar tendinopathy; in terms of lighter colored clusters, the trends in the field were different platelet-rich plasma concentration, pilot study, articular cartilage avulsion, musculoskeletal lesion, and collagen hydrogel.

### 5.3. Trend analysis of research frontiers based on the emergence of key literature

The citations represent key literature that has been frequently cited over a period of time, thus indicating hot spots and trends, Figure 7D. Among the 25 most frequently cited citations screened by the Web of Science database, the strongest mutation was found by de Vos RJ in 2010, who found that PRP injections did not lead to greater improvement in pain and activity compared to saline injections in patients with chronic achilles tendinopathy treated with centrifugal exercise (41.18), followed by Sanchez M in 2007, who found that the use of a platelet-rich fibrin matrix did not lead to greater improvement in pain and activity. In third place, de Mos M in 2008 found that platelet-rich clot releasers (PRCR) stimulated cell proliferation and total collagen production. Proliferation and total collagen production slightly increased the expression of matrix degrading enzymes and endogenous growth factors. Platelet-rich plasma was found to enhance tendon repair from a cell culture perspective (32.79). The literature can be broadly divided into four parts based on the emergence of the literature, the first part focusing on the *in vitro* cellular molecular biology of platelet-rich plasma and its preparations on tendon and ligament cells (2003–2014) (33, 40–42) The second part focuses on the comparative efficacy of platelet-rich plasma and its preparations on specific tendon and ligament injuries (chronic epicondylitis, rotator cuff repair, jumper's knee, chronic achilles tendinopathy) (based on intention-to-treat principles) and longer-term follow up studies (≥1 year) after treatment (2011–2016) (30, 35, 43); the third part focuses on a large sample size double-blind clinical randomized controlled study of the efficacy of platelet-rich plasma and its preparations in tendon injuries (2014–2019) (27, 44); Part IV focuses on a meta-analysis of the efficacy of platelet-rich plasma on tendon and ligament healing and an investigation of the efficacy of sequential multiple injections of PRP on tendon or ligament healing (2017-present) (18, 45) Based on a careful reading of the high-burst citations, it is possible to foresee future research trends exploring the efficacy of platelet-rich plasma and its preparations on tendon and ligament healing, longer follow up, network meta-analyses based on a large body of literature, studies on the efficacy of multiple consecutive injections of PRP on tendon or ligament healing, the role of PRP in acute, chronic and Severe tendon and ligament healing, comparative studies of the optimal concentration of PRP for injection time or residence time at the site of injury, studies of standardized doses and composition of PRP and formulations, and studies of the effects of injectable agents and injection site PH.

### 5.4. Analysis of research hotspots and frontier trends based on thematic maps

**Figure 8:**
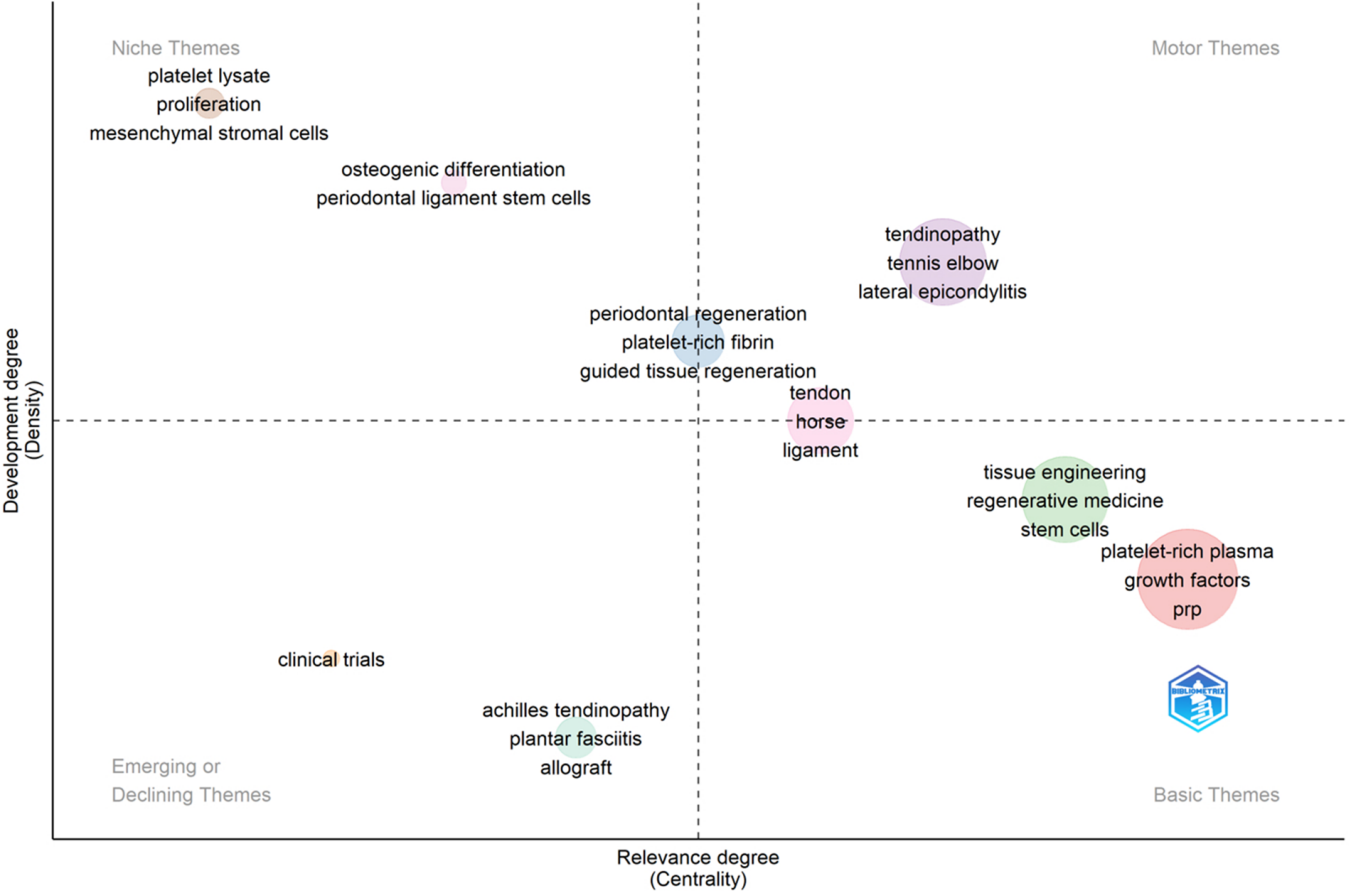
Thematic map of using platelet-rich plasma to treat ligament and tendon injuries.

A two-dimensional matrix is used to illustrate the thematic maps created with the R-Bibliometrix software. The x-axis and y-axis, respectively, indicate the two dimensions of matrix centrality and density. The y-axis represents density, or the centrality of the subject, while the X-axis represents centrality, or the salience of the subject. Thus, the upper right quadrant refers to motor themes that are both important and well developed, the upper left quadrant refers to niche themes that have been well developed but are not important to the current field, the lower left quadrant refers to emerging or declining themes that are not well developed and may have just emerged and perhaps are about to disappear, and the lower right quadrant refers to basic topics that are important to the field but have not been well developed. It generally refers to the basic themes, Figure 8. One can find one keyword bubble for tendinopathy and lateral epicondylitis in the motor theme quadrant; two keyword bubbles for platelet lysate, proliferation, mesenchymal stromal cells and osteogenic differentiation, periodontal ligament stem cells in the niche theme quadrant; two keyword bubbles for clinical trials and achilles tendinopathy, plantar fasciitis, allografts in the emerging or declining theme quadrant; and two keyword bubbles of tissue engineering, regenerative medicine, stem cells, and platelet-rich plasma, growth factors, PRP in the basic theme quadrant; the keyword bubble of periodontal regeneration, platelet-rich fibrin, and guided tissue regeneration spanning both the motor theme and the niche theme; and the keyword bubble of tendon, horse, and ligament spanning both the motor theme and the basic theme. Based on the quadrant where the key bubbles are located, it can be speculated that tendinopathy, lateral epicondylitis, horses, guided tissue regeneration, and ligaments are the hotspots of research in this field; while achilles tendinopathy, plantar fasciitis, allograft, and clinical trial themes may be developed or disappear in the future, while in the direction of tissue engineering, regenerative medicine, horses, stem cells and platelet-rich plasma, growth factors, and PRP themes need to be more in-depth research.

## 6. Discussion

### 6.1. Platelet-rich plasma is widely used in the treatment of tendon ligament injuries

Since its discovery, platelet-rich plasma has been widely used in the fields of dentistry, orthopedics, ophthalmology, and otorhinolaryngology, especially in the direction of orthopedics and sports medicine research gradually increased, often applied to fracture non-healing, tendon and ligament injuries, osteoarthritis, muscle injuries, etc., especially in refractory tendinopathy and ligament degenerative lesions, with better clinical efficacy (46, 47).

Platelet-rich plasma injections are a common clinical treatment for chronic tendinopathies. According to statistics, approximately more than 86,000 athletes in Europe and the United States receive platelet-rich plasma treatment for tendon, ligament, or muscle injuries each year (48). The reason for this is that platelet-rich plasma is thought to contain concentrated amounts of a variety of growth factors, including platelet-derived growth factor, transforming growth factor beta and insulin-like growth factor, which have the ability to promote tendon healing (49, 50). Platelet-rich plasma is largely prepared from the patient's own plasma in clinical applications and has an excellent safety profile, with few adverse effects in published randomized controlled clinical trials (51). Many platelet-rich plasma kits are commercially available for use in orthopedic clinics. In addition, platelet-rich plasma is non-invasive in its injectable form and can be easily administered in the clinical setting (16). Researchers have mostly studied the efficacy of platelet-rich plasma in tendinopathies using a randomized controlled clinical trial approach, with a particular focus on the treatment of humeral epicondylitis (35) ZHANG et al. (52) used data from United States. health insurance agencies to count patients treated with platelet-rich plasma injections across the United States between 2010 and 2011 and found that the main tendinopathies treated with platelet-rich plasma injections included rotator cuff injuries, humeral epicondylitis, and plantar fasciitis.

Platelet-rich plasma is often used in combination therapy for tendon ligament injuries and is often combined with physiotherapy based on a centrifugal exercise in platelet-rich plasma injections for rotator cuff injuries (31), humeral epicondylitis (30), Achilles tendinopathy (22), and patellar tendinopathy (36), with good clinical outcomes. Platelet-rich plasma combined with stenting techniques to promote ACL injury repair has also been studied to a small extent (25). In recent years, platelet-rich plasma combined with mechanical stimulation for tendon ligament injuries has gradually gained attention. Currently, animal studies have shown that mechanical stimulation combined with platelet-rich plasma can effectively promote early healing of the bony-tendon interface after rotator cuff injury (53).

### 6.2. Effectiveness of platelet-rich plasma for tendon ligament injuries is influenced by several factors

Although platelet-rich plasma is widely used in the treatment of tendon ligament injuries, there are many conflicting results reported on the therapeutic effects of platelet-rich plasma.DRAGOO et al. (8) Injected platelet-rich plasma combined with centrifugal exercise accelerated the recovery of patellar tendinitis in patients with patellar tendinitis.BOESEN et al. (54) evaluated patients' pain scores (VISA-A) before and 6, 12, and 24 weeks after injection, and found that platelet-rich plasma combined with centrifugal training for chronic achilles tendinopathy was more effective in reducing pain, improving mobility, reducing tendon thickness and intra-tendon vascular density. In contrast, de Vos RJ et al. (24) used a randomized control method to randomize 54 patients (>2 months duration) with chronic mid-portion achilles tendinopathy into two groups after platelet-rich plasma or saline injection combined with 12 weeks of centrifugal exercise training. Patient pain scores (VISA-A) were assessed before and 6, 12, and 24 weeks after injection, and showed that platelet-rich plasma injection did not improve patient pain scores compared with placebo. For this contradictory result to occur, it may be closely related to some influencing factors.

#### 6.2.1. Non-uniformity in preparation methods and composition of platelet-rich plasma and its related preparations

Since platelet-rich plasma has been widely used in the treatment of tendinopathies, there has been controversy regarding its preparation standards. Different platelet-rich plasma preparation systems differ in terms of equipment materials, the volume of whole blood used for centrifugation, anticoagulation method, speed, number and time during centrifugation, and separation method of each group after centrifugation. Therefore, the composition of platelet-rich plasma preparations produced by different preparation systems has a large variation (55, 56). For example, there were significant differences in platelet-rich plasma prepared by three different systems, Cascade, Magellan, and Biomet GPS III, in terms of platelet capture efficiency, leukocyte concentration, platelet-derived growth factor AB, platelet-derived growth factor BB, and vascular endothelial growth factor concentration (57). Currently, there are no uniform standards for platelet, leukocyte and related factor concentrations in platelet-rich plasma, and most experiments tend to mention only the brand of platelet-rich plasma preparation kits without detailing the specific parameters of the extracted platelet-rich plasma (58) The specific parameters of platelet-rich plasma extraction are not detailed. Currently, a PRP platelet count of 1 million/*μ*l measured in a standard 6 ml aliquot has become the benchmark for “therapeutic PRP (29). In addition, classification systems exist for analyzing platelet-rich plasma (45, 55). Among them, DELONG et al. (56) The PAW (platelet, activation, white blood cells) system created by DELONG et al. is considered to be a reasonable classification system for platelet-rich plasma and provides a relatively accurate comparison of the therapeutic effect of platelet-rich plasma with different component concentrations on tendinopathies by evaluating the absolute platelet count, activation mode, and leukocyte and neutrophil concentrations of platelet-rich plasma.

Platelet-rich plasma can be prepared by plasma exchange and density-gradient centrifugation, the former using automatic separation equipment for medical blood components, with a higher concentration and purity of platelets in the preparation, which is essentially free of red and white blood cells and is a platelet-rich plasma preparation with a lack of white blood cells. Compared with the density gradient centrifugation method, the plasma separation and replacement method can achieve rapid preparation of platelet-rich plasma, and the concentration of each component of platelet-rich plasma is more stable, but the expensive equipment system and high operating technology threshold limits its widespread application, most of the relevant basic experiments and clinical research still use the traditional density gradient centrifugation method to prepare platelet-rich plasma. The density gradient centrifugation method is simple, easy to use and inexpensive. The mainstream density gradient centrifugation methods include the plasma layer method and the white film layer method. The former preparation is basically free of red blood cells and leukocytes and has a low platelet content of 1.5–3 times the baseline level, making it a leukocyte-deficient platelet-rich plasma preparation. The latter preparation usually has a higher platelet content, 3–8 times the baseline level, and a higher leukocyte content, making it a leukocyte-rich platelet-rich plasma preparation (37).. The leukocyte-rich platelet-rich plasma produced from the latter can be filtered out to produce platelet-rich plasma with a high platelet concentration of leukocytes (59). A variety of commercial platelet-rich plasma preparation systems are available, which can be divided into low yield and high yield systems based on platelet concentration: low yield systems include Arthrex ACP, Cascade PPRtherapy, PRGF, RegenKit-BCT, and high yield systems include BiometGPS, Harvest SmartPRep 2 APC+, Arterio (60). However, current clinical trials generally need more detailed documentation of platelet-rich plasma preparation protocols and formulation components to reproduce experiments or to compare differences in the efficacy of different components of platelet-rich plasma (61). For example, in a meta-analysis assessing the use of platelet-rich plasma in the musculoskeletal field, only 11 (10%) of the 105 clinical studies included provided a comprehensive report of the platelet-rich plasma preparation protocol, and 17 (16%) provided a report of the composition of the platelet-rich plasma preparation (55).

The optimal platelet concentration of platelet-rich plasma in the treatment of tendinopathies has yet to be discovered. It is currently believed that low platelet concentration platelet-rich plasma is of limited therapeutic benefit in tendinopathies. The effectiveness of platelet-rich plasma of moderate platelet concentration, produced by the plasma layer method, in the treatment of tendinopathies has been demonstrated in numerous trials (40, 62, 63). Platelet-rich plasma preparations with excessive platelet concentrations may lead to local apoptosis, downregulation of growth factor receptor numbers and desensitization, thereby inhibiting the therapeutic effect on tendinopathies (64). In addition, different preparation systems produce platelet-rich plasma with different proportions of neutrophils, lymphocytes, and monocytes (65). The role of leukocytes is controversial, and some authors believe that leukocytes in platelet-rich plasma have a positive effect, with leukocytes having an antimicrobial effect and modulating the local immune environment, helping to prevent or control infection in damaged tendons (57) In addition, leukocytes further increase the concentration of growth factors in platelet-rich plasma by releasing growth factors or stimulating platelets to release growth factors (66). Another group of scholars has argued that leukocytes are not an ideal component of platelet-rich plasma and that the release of collagenases such as matrix metalloproteinase 8 and matrix metalloproteinase 6 by leukocytes has a detrimental effect on tendon matrix deposition (67, 68).

#### 6.2.2. Diversity of activation modes for platelet rich plasma

The prepared platelet-rich plasma can maintain an anticoagulant state for more than 8 h. However, platelet-rich plasma must be activated to release the platelet granule contents for therapeutic action (69).. There are five main activation methods for platelet-rich plasma: ① the use of bovine thrombin, but activated platelet-rich plasma releases growth factors too quickly and with limited bioactivity, risking coagulopathy in patients and is not recommended. ② Using calcium chloride solution, the activated platelet-rich plasma maintains a high concentration of growth factors for a long time, but the level of platelet activation is low and the release of growth factors is slow (61). ③ The autologous thrombin preparation kit method, which has the advantages of high safety and short activation time, is a new activation method that combines the thrombin method and the calcium chloride solution method (70). ④ Endogenous type I collagen method, with the advantages of high growth factor release and inhibition of fibrin clot contraction, is a safer and more effective activation method than bovine thrombin (56). The effectiveness of platelet-rich plasma activation has been demonstrated in basic tests, providing ideas for future clinical application (71). ⑤ Photoactivation is a relatively new method, an alternative method to activating platelets, with the advantages of high safety, long-term, and high release of growth factors. Therefore, different activation methods can have an impact on the test results (72).

#### 6.2.3. Platelet-rich plasma treatment effect is closely related to the type of tendon ligament injury

Platelet-rich plasma injections are considered an effective treatment for humeral epicondylitis. Several publications have reported significant short-term and long-term efficacy of platelet-rich plasma for its treatment (73, 74). MISHRA et al. (44) evaluated 230 patients with humeral epicondylitis who had failed physical therapy with leukocyte-rich platelet-rich plasma and found a 71.5% improvement in pain scores 24 weeks after treatment. Platelet-rich plasma provided longer sustained symptom relief than corticosteroid injections for humeral epicondylitis and therefore had a more sustainable treatment effect. gosens et al. (35) and PEERBOOMS et al. (30) evaluated the efficacy of leukocyte-rich platelet-rich plasma vs. corticosteroids in 100 patients with a history of intractable chronic epicondylitis (at least 6 months) who had failed to respond to conservative treatment, showing that patients treated with platelet-rich plasma continued to report symptom relief 1 year after injection, whereas the short-term efficacy of corticosteroids began to decline after 12 weeks and patients treated with platelet-rich plasma had sustained symptom improvement at 2 years. The results of platelet-rich plasma in achilles tendinopathy have been variable (75). analyzed 24 articles on platelet-rich plasma for achilles tendinopathy and found that all uncontrolled studies showed good clinical outcomes of platelet-rich plasma for achilles tendinopathy, restoring motor function and maintaining efficacy over time, but the results of double-blind controlled studies did not show that platelet-rich plasma was superior to other treatments for achilles tendinopathy (24) Sanchez M et al. (23) In 12 athletes with complete achilles tendon tears who received open suture repair combined with a platelet-rich fibrin matrix (PRGF) preparation, it was found that athletes who received PRGF returned to the range of motion and low-intensity running earlier and resumed training activities with less increase in the cross-sectional area of the repaired tendon; the results of platelet-rich plasma for rotator cuff injuries were variable. Castricini R et al. (31) found that the use of PRFM did not improve the healing of small to medium-sized rotator cuff tears repaired with double-row anchor staples, while Randelli *P* et al. (32) in 53 patients with complete rotator cuff tears repaired with single-row anchor staple sutures combined with PRP, found that autologous PRP reduced pain in the first few months postoperatively, and long-term results in subgroups of grade 1 and 2 tears suggested that PRP had a positive effect on the recovery of rotator cuff external rotation strength. Platelet-rich plasma has been understudied for other tendon ligament injuries. Only a very small number of high-quality studies support the efficacy of using leukocyte-rich platelet-rich plasma and multiple sequential injections of platelet-rich plasma for the treatment of chronic intractable patellar tendinopathy (8, 76). For other tendon ligament injuries such as anterior cruciate ligament (25), plantar fasciitis (77), gluteal fasciitis (78), and stenosing tenosynovitis (79) However, there is insufficient evidence that platelet-rich plasma has a significant therapeutic effect on these diseases, as the number of cases studied is insufficient (80).

#### 6.2.4. Influence of injection time, injection site, administration method, number of administrations, acidity and evaluation method

It has been shown that receiving platelet-rich plasma injections in the early stages of tendinopathy is more effective than late treatment (81). The site of disease, for example, mid tendinopathy and lower tendinopathy, which are common in achilles tendinopathy, also differs when treated with platelet-rich plasma (54). Tendinopathy is better treated with multiple consecutive injections of platelet-rich plasma than with a single injection (36). The Peppering technique, a multi-point injection method, has the advantage of eliminating a wide range of inflammation (82). In recent years, a variety of platelet-rich plasma delivery systems have been successfully developed to achieve prolonged local effects, including injectable hydrogel microspheres (83), injectable hydrogel formulations (84), and so on. The drug delivery system has been successfully developed. Platelet-rich plasma injections are more effective when the pH of the injection site is acidic than when the pH is alkaline (29). In addition, a variety of platelet-rich plasma is often used in clinical studies. In addition, various scales are often used in clinical studies as indicators of treatment effectiveness, including visual analog scores, PRTEE, DASH, and VISA-A scores, which are based on the patient's own assessment of pain intensity and tendon function and are therefore inevitably highly subjective and highly variable. If the results of the scales are to be objective, clinical studies should use a larger number of patients to assess the effects of platelet-rich plasma therapy. However, in the current clinical studies, many of the sample sizes are relatively small. In addition to the disease itself and the efficacy evaluation system, there is variability in patient age, gender, treatment history, and activity level, all of which may reduce the statistical power to detect efficacy (85), all of which may be factors affecting the evaluation of the efficacy of platelet-rich plasma in the treatment of tendinopathies.

In general, the effectiveness of platelet-rich plasma in the treatment of tendon ligament injuries is influenced by several factors, the main ones being the inconsistency in the preparation of platelet-rich plasma and its related preparations and components, which can significantly affect the efficacy; secondly, the five different activation methods of platelet-rich plasma can also lead to differences in efficacy. The effectiveness of platelet-rich plasma in the treatment of tendon ligament injuries cannot be generalized, and the clinical efficacy varies for different injury sites. In addition, the efficacy of platelet-rich plasma in the treatment of tendon ligament injuries is also influenced by the time of injection, injection site, mode of administration, number of administrations, acidity, and evaluation methods.

### 6.3. Molecular biological mechanisms of platelet-rich plasma for tendinopathy treatment are gaining attention

The research hotspot regarding platelet-rich plasma in the treatment of tendinopathies since 2003 has transitioned from the study of the effect of platelet-rich plasma on disease treatment alone to the study of the biological mechanisms of platelet-rich plasma in tendinopathies. Platelet-rich plasma contains many different growth factors and cytokines. Numerous *in vitro* and animal model studies have shown that platelet-rich plasma positively affects tendon healing by releasing these bioactive proteins to promote collagen expression, angiogenesis and cell migration, differentiation, and proliferation and to increase extracellular matrix production. Platelet-rich plasma contains cytokines that activate multiple cellular transduction pathways, and some of these signaling molecules located downstream of individual receptors can inhibit or further enhance the activation of other pathways regulated by different receptors. For example, in mediating inflammation for pain relief, platelet-rich plasma contains interleukin 1*β* and interleukin 10, which are known to be inflammation-activating factors in intracellular signaling pathways, and the interleukin 10 signaling pathway can inhibit interleukin 1*β*-activated pathways and reduce the expression of pro-inflammatory genes (86). ZHANG (87) found that hepatocyte growth factor in platelet-rich plasma antagonized interleukin 1*β* and inhibited the expression of cyclooxygenase 1, cyclooxygenase 2, prostaglandin E2 synthase genes and prostaglandin E2 production in patellar tendon cells, thereby modulating the local inflammatory response and reducing pain. In addition, hepatocyte growth factor in platelet-rich plasma inhibits the inflammatory response of the diseased tendon by increasing the expression of I*κ*B*α* (88). In terms of promoting stem cell migration, differentiation, and cellular proliferation, platelets secrete platelet-derived growth factor B, fibroblast growth factor *β*, chemokine CXCL (89), VEGF, endothelial growth factor, hepatocyte growth factor (40), and many other cytokines, which can provide the initial signal for stem cell migration, increase circulating system-derived cells in tendons, and promote cellular behaviors such as proliferation and differentiation. The activation of signaling pathways and cellular responses in platelet-rich plasma during the treatment of tendinopathies still needs to be studied in more depth.

## 7. Conclusion

This study provides the first bibliometric and visual analysis of international research on using platelet-rich plasma to treat ligament and tendon injuries over the past 20 years from multiple perspectives, providing a new perspective for rapid understanding of the field of using platelet-rich plasma to treat ligament and tendon injuries. Based on annual publication volume and trends, the United States and China will continue to dominate in terms of publication volume, with some collaboration among high-impact authors and further collaboration still needed in different countries and institutions. Research hotspots focus on tennis elbow, ACL, rotator cuff repair, achilles tendon, mesenchymal stem cells, guided tissue regeneration, network meta-analysis, chronic patellar tendinopathy, and follow up. Sodium hyaluronate, clinical outcomes, mesenchymal stem cells, rotator cuff tears, growth factors, and follow up can represent research trends. Currently, platelet-rich plasma is widely used in the treatment of tendon ligament injuries. Its clinical efficacy is affected by several factors, the main ones being the non-uniformity of the preparation methods and components of platelet-rich plasma and its related preparations, and it is suggested that subsequent studies should establish a more reasonable classification system for platelet-rich plasma. Different activation methods of platelet-rich plasma may also lead to differences in efficacy. In addition, clinical efficacy is also influenced by factors such as injection time, injection site, administration method, number of administrations, acidity, and evaluation methods. Therefore, it is essential to establish uniform and reasonable preparation standards and usage guidelines in the future. The effectiveness of platelet-rich plasma in the treatment of tendon and ligament injuries cannot be generalized, and its applicability to different injury diseases remains controversial. The molecular biology of platelet-rich plasma for tendon ligament therapy is increasingly being emphasized, but the molecular biology of the pathways through which platelet-rich plasma affects tendon ligament healing is still inconclusive. It is recommended that more in-depth research in basic molecular biology, especially *in vitro* experiments and animal models, should be conducted.

## Data Availability

The raw data supporting the conclusions of this article will be made available by the authors, without undue reservation.
