# Supplementary figures and images for: Global research trends on platelet-rich plasma for tendon and ligament injuries from the past two decades: A bibliometric and visualized study
Source: Front Surg. 2023 Feb 10;10:1113491. doi: 10.3389/fsurg.2023.1113491 (PMC9950278; doi:10.3389/fsurg.2023.1113491)

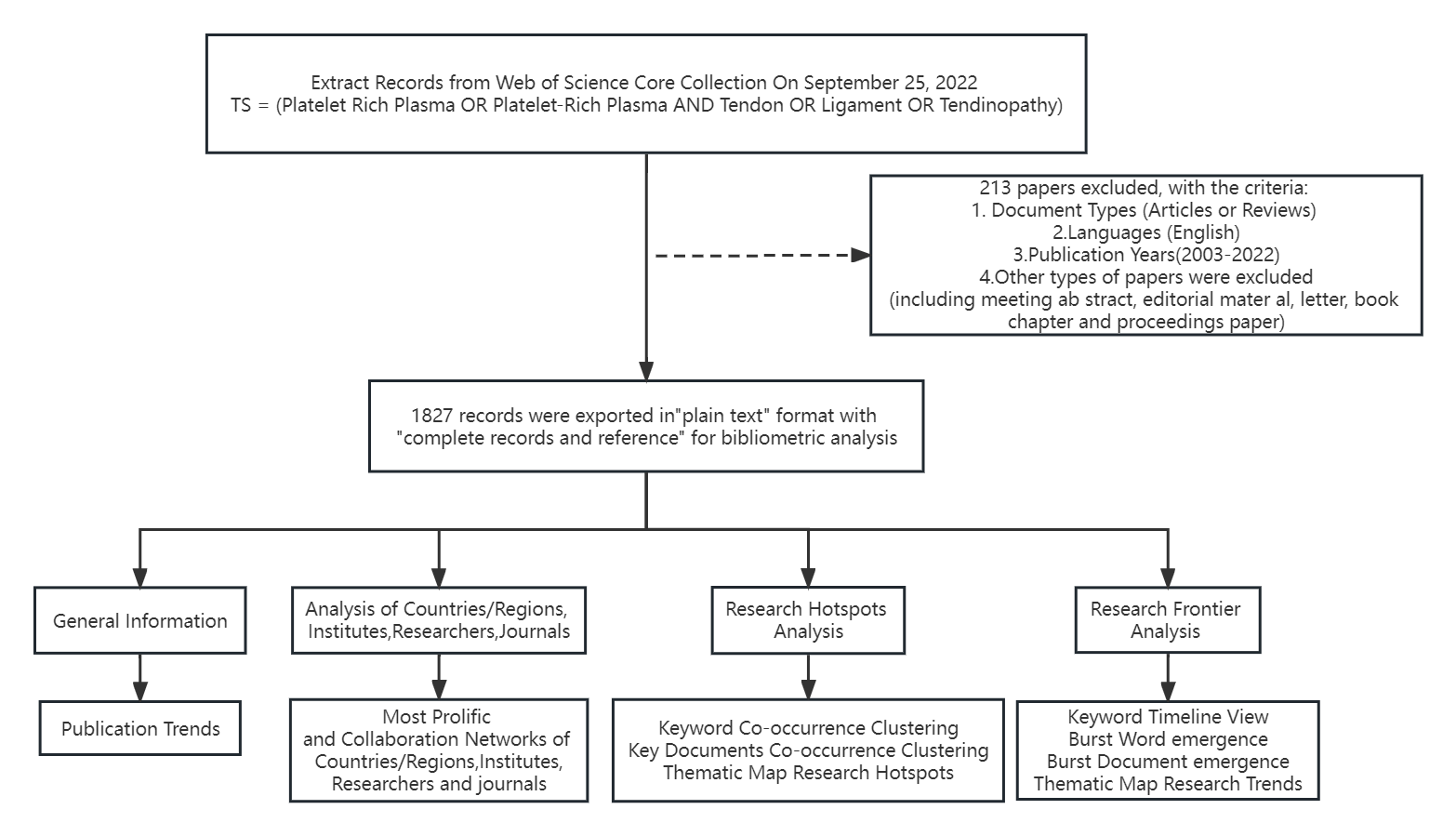

Supplement: Supplementary file 1 [file Datasheet1.zip › Data Sheet 1_v1/Figure/Figure 1.jpg]

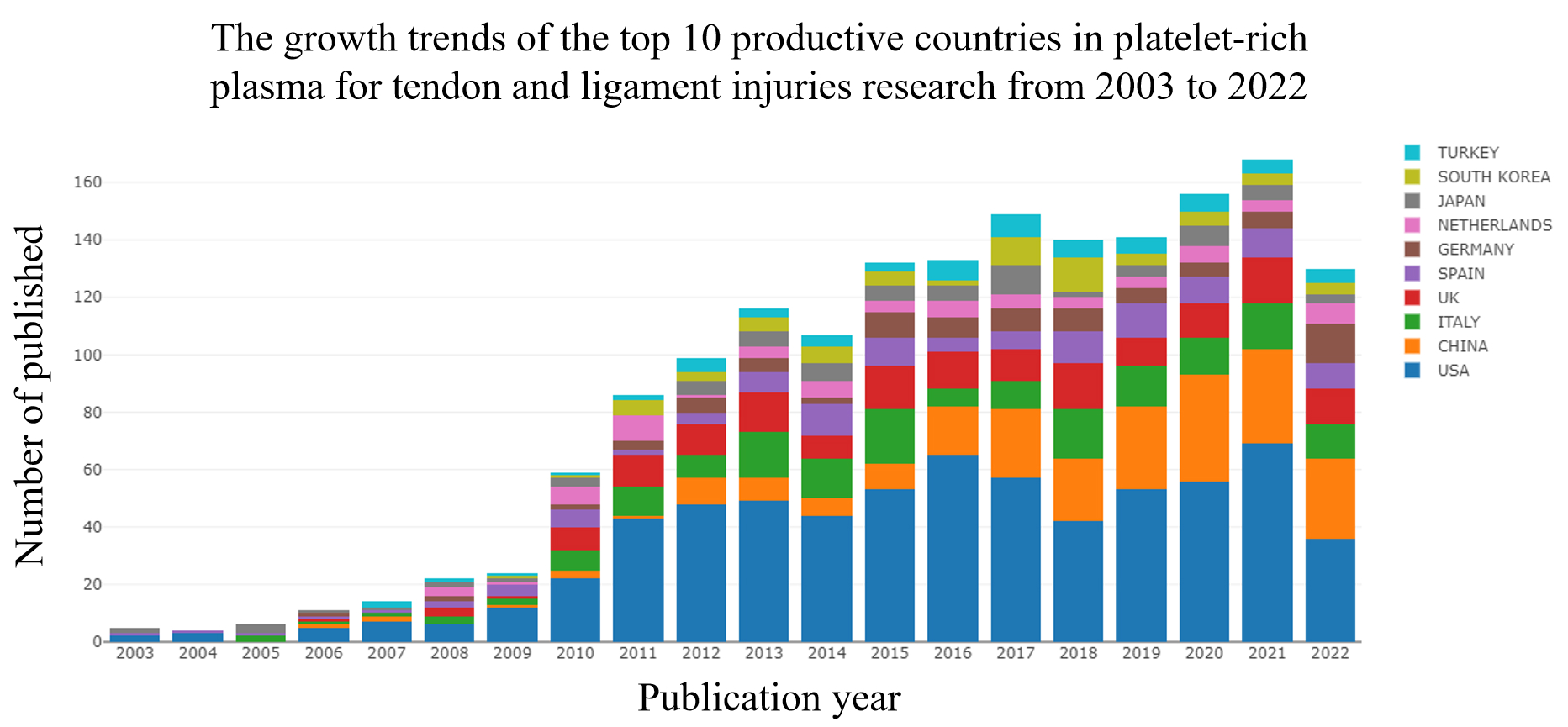

Supplement: Supplementary file 1 [file Datasheet1.zip › Data Sheet 1_v1/Figure/Figure 2(A).jpg]

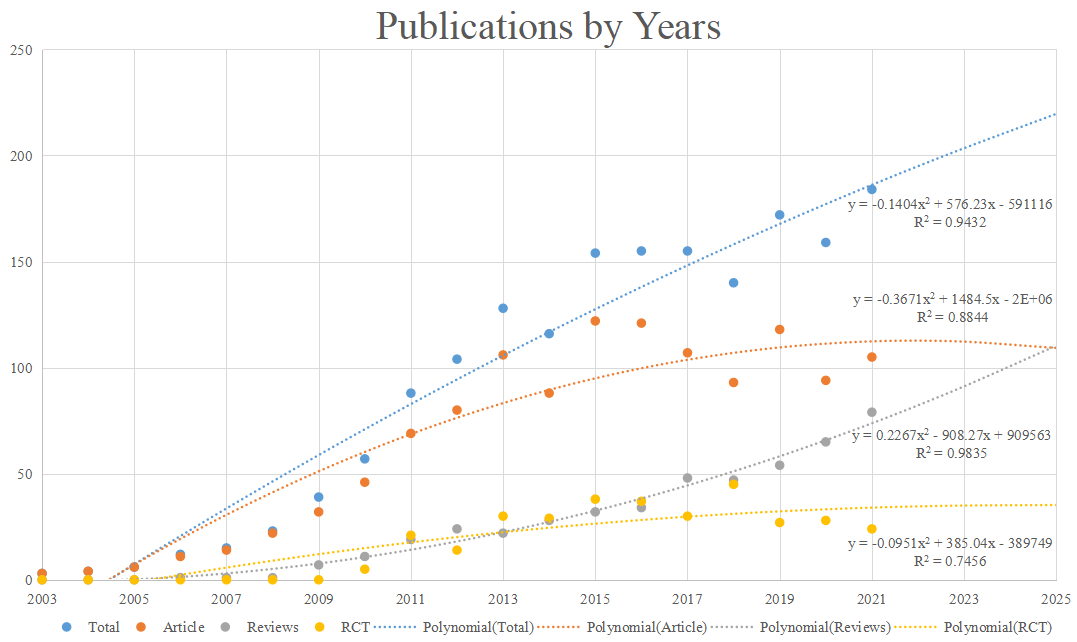

Supplement: Supplementary file 1 [file Datasheet1.zip › Data Sheet 1_v1/Figure/Figure 2(B).jpg]

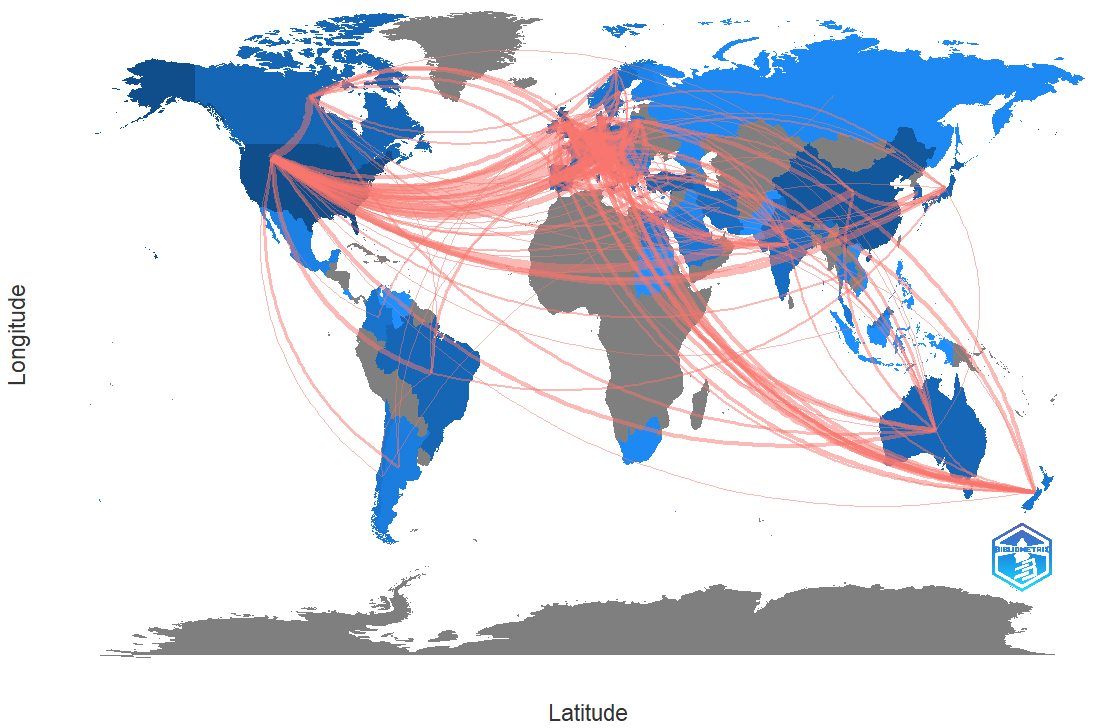

Supplement: Supplementary file 1 [file Datasheet1.zip › Data Sheet 1_v1/Figure/Figure 2(C).jpg]

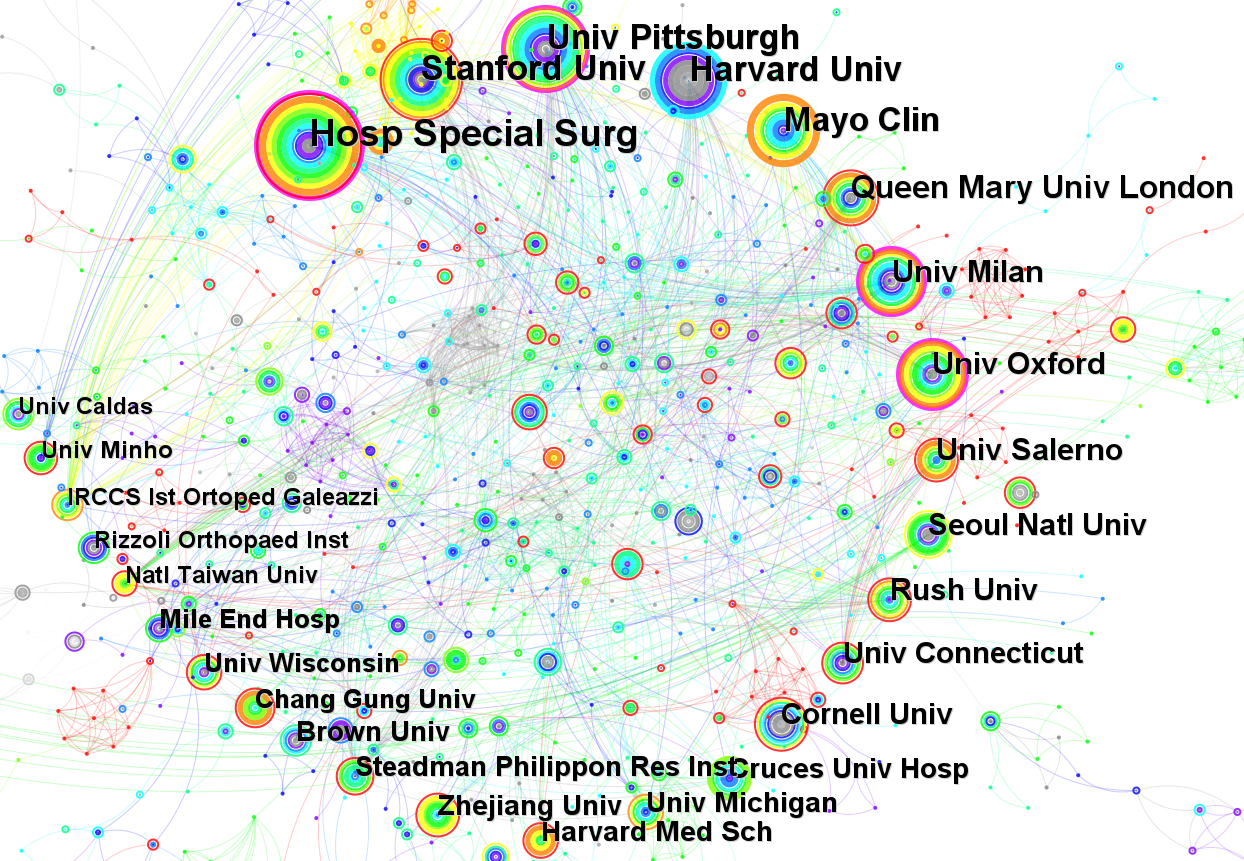

Supplement: Supplementary file 1 [file Datasheet1.zip › Data Sheet 1_v1/Figure/Figure 2(D).jpg]

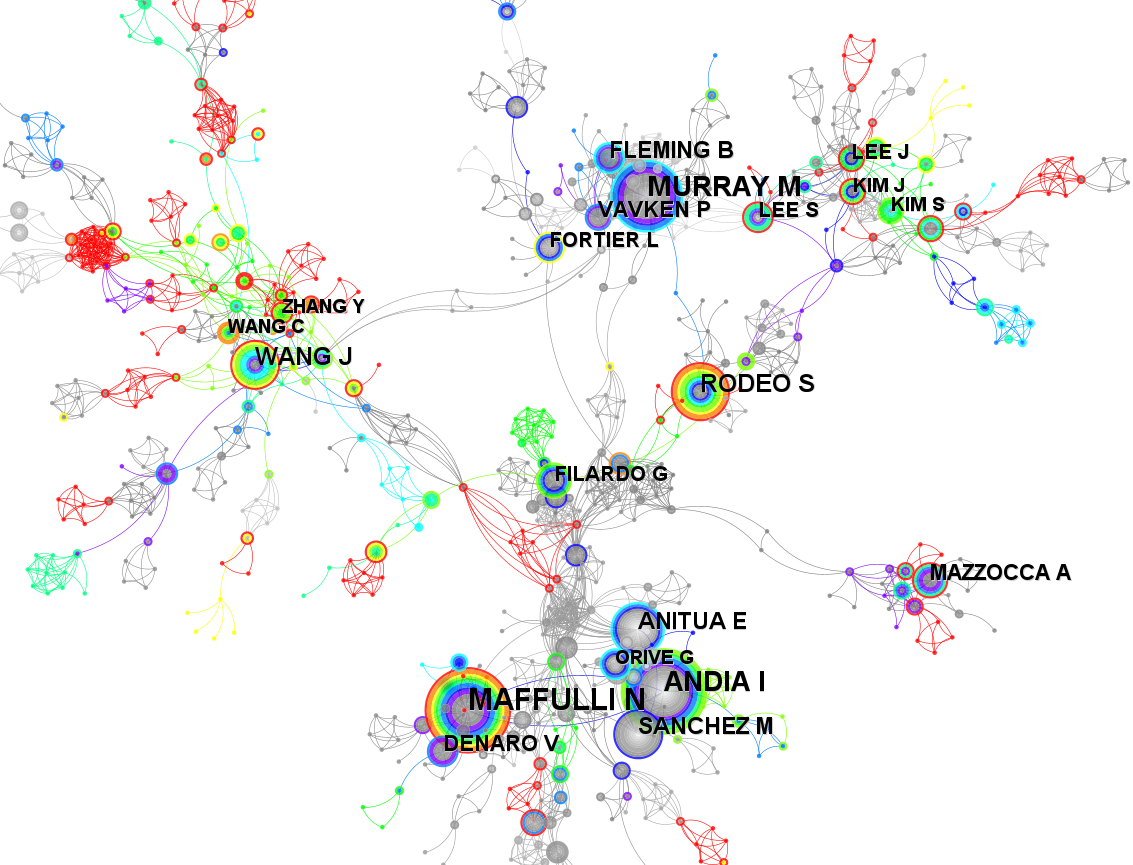

Supplement: Supplementary file 1 [file Datasheet1.zip › Data Sheet 1_v1/Figure/Figure 3(A).jpg]

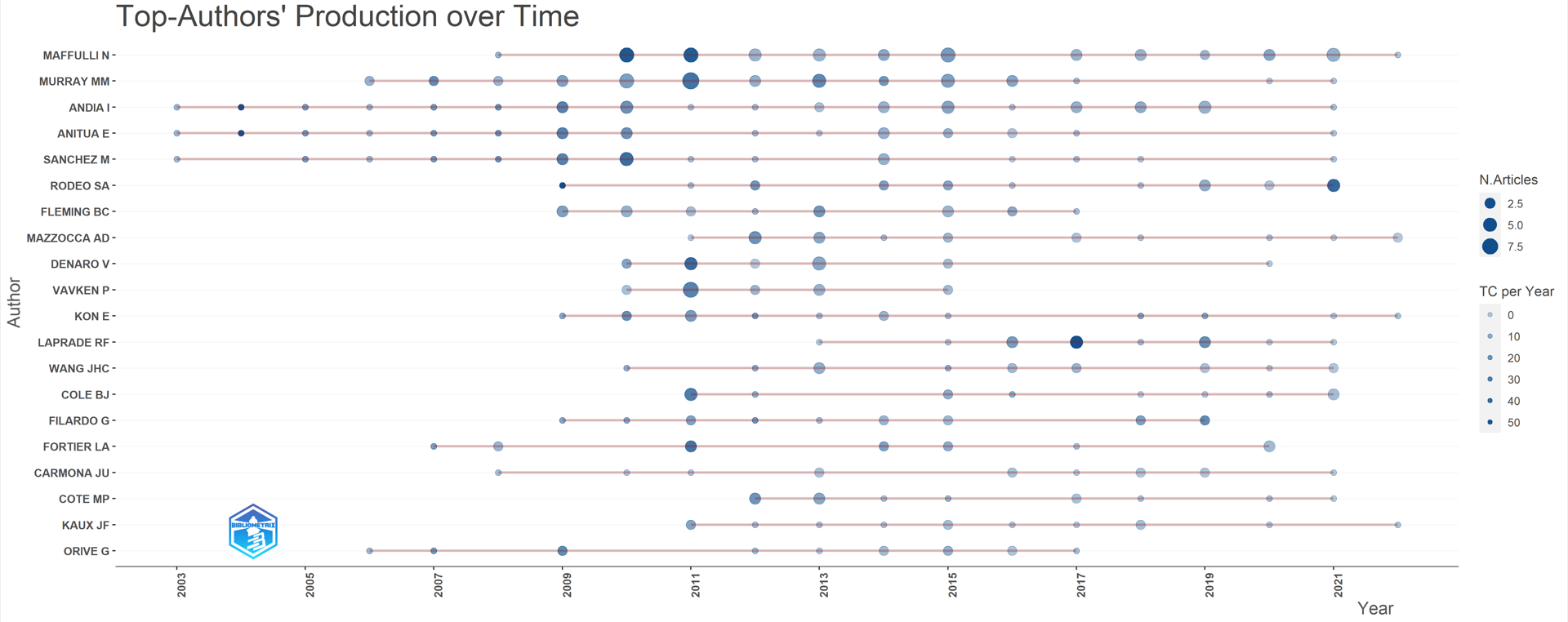

Supplement: Supplementary file 1 [file Datasheet1.zip › Data Sheet 1_v1/Figure/Figure 3(B).jpg]

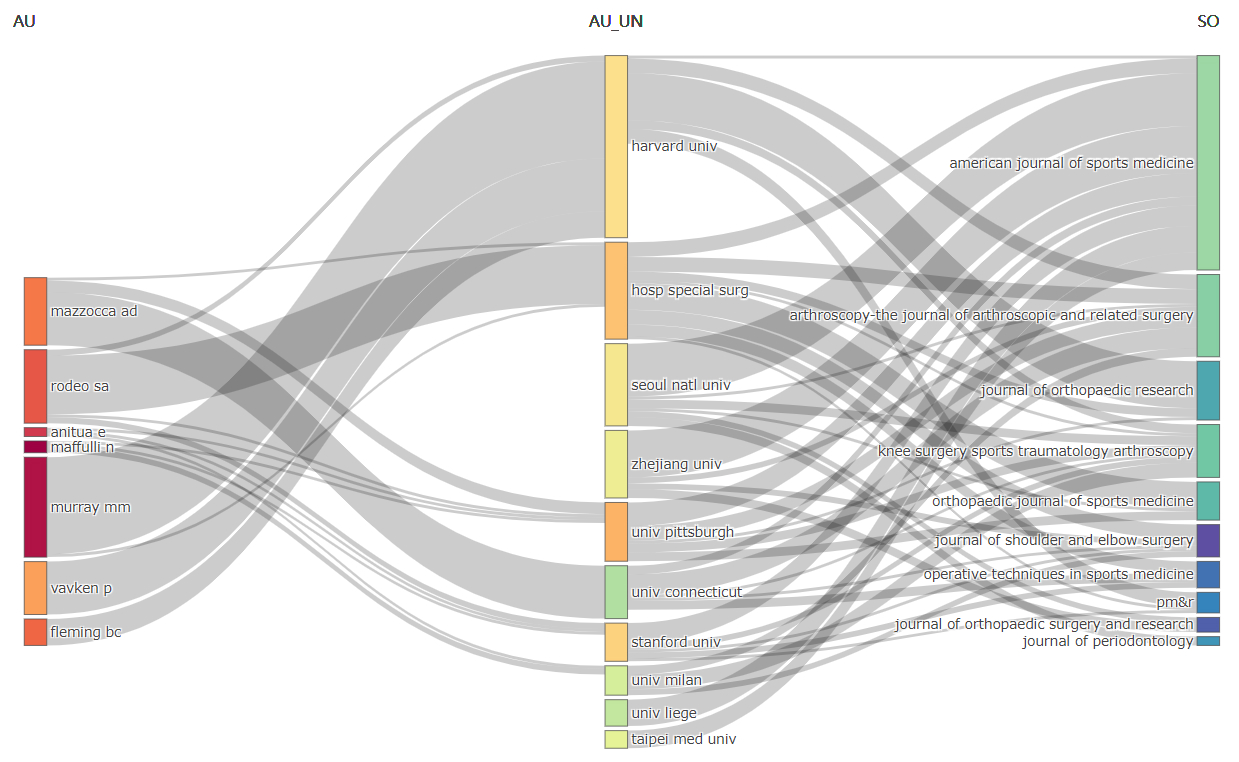

Supplement: Supplementary file 1 [file Datasheet1.zip › Data Sheet 1_v1/Figure/Figure 3(C).jpg]

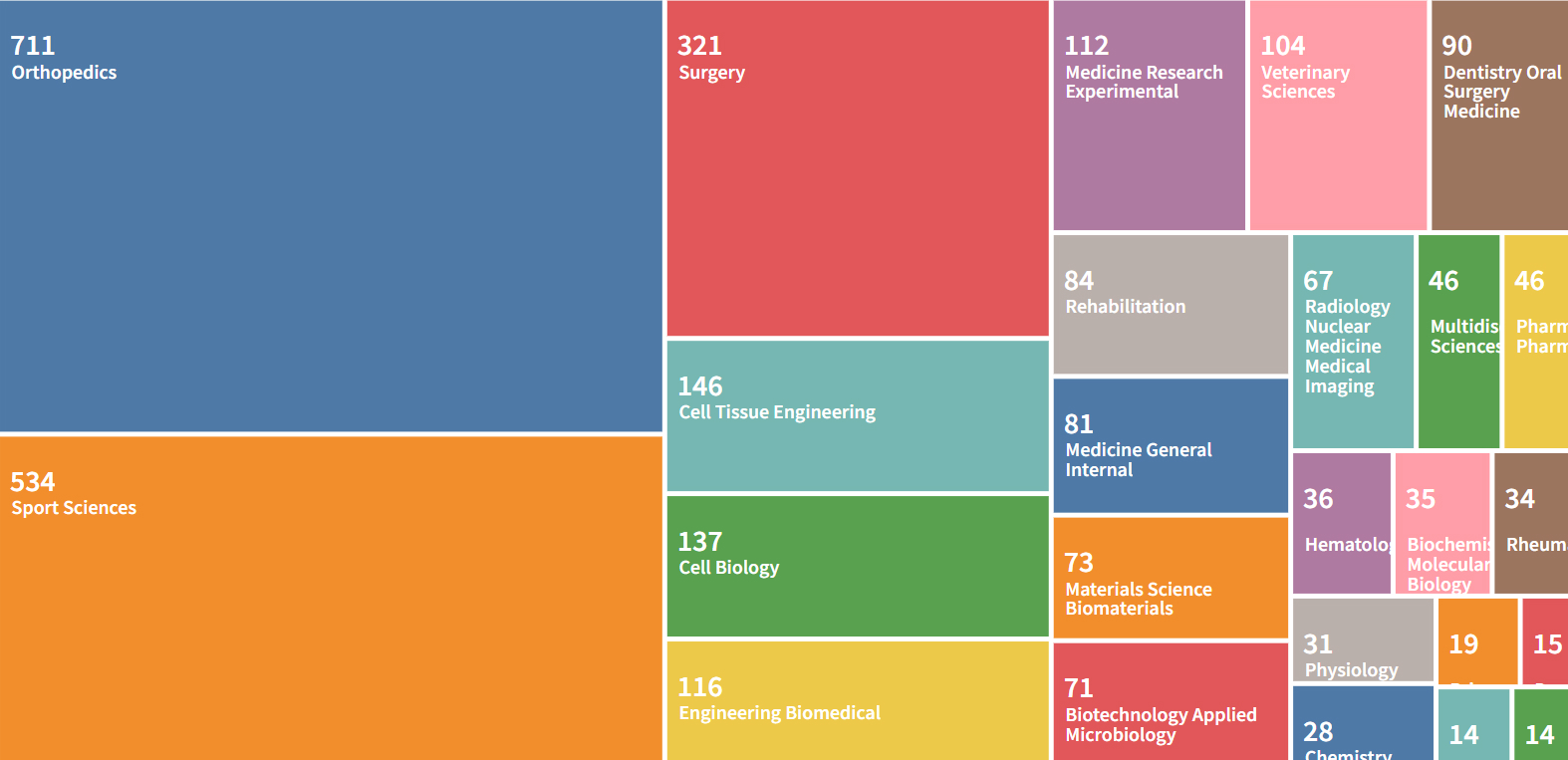

Supplement: Supplementary file 1 [file Datasheet1.zip › Data Sheet 1_v1/Figure/Figure 3(D).jpg]

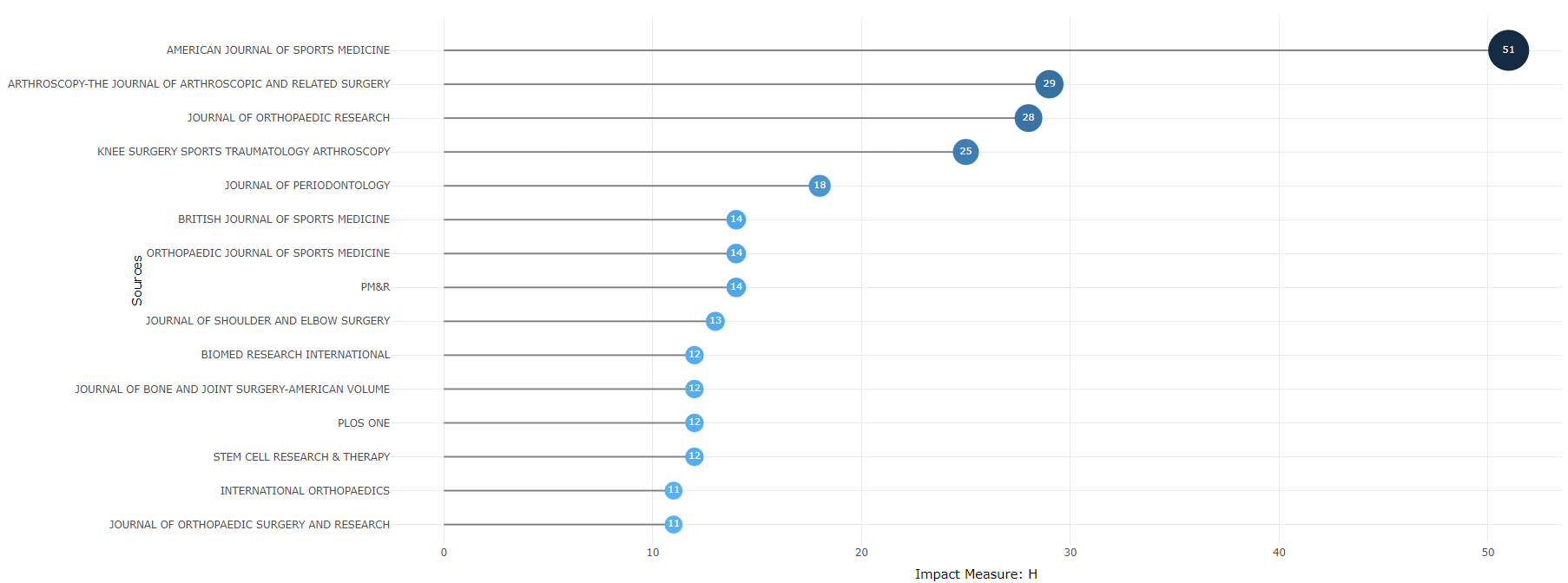

Supplement: Supplementary file 1 [file Datasheet1.zip › Data Sheet 1_v1/Figure/Figure 4(A).jpg]

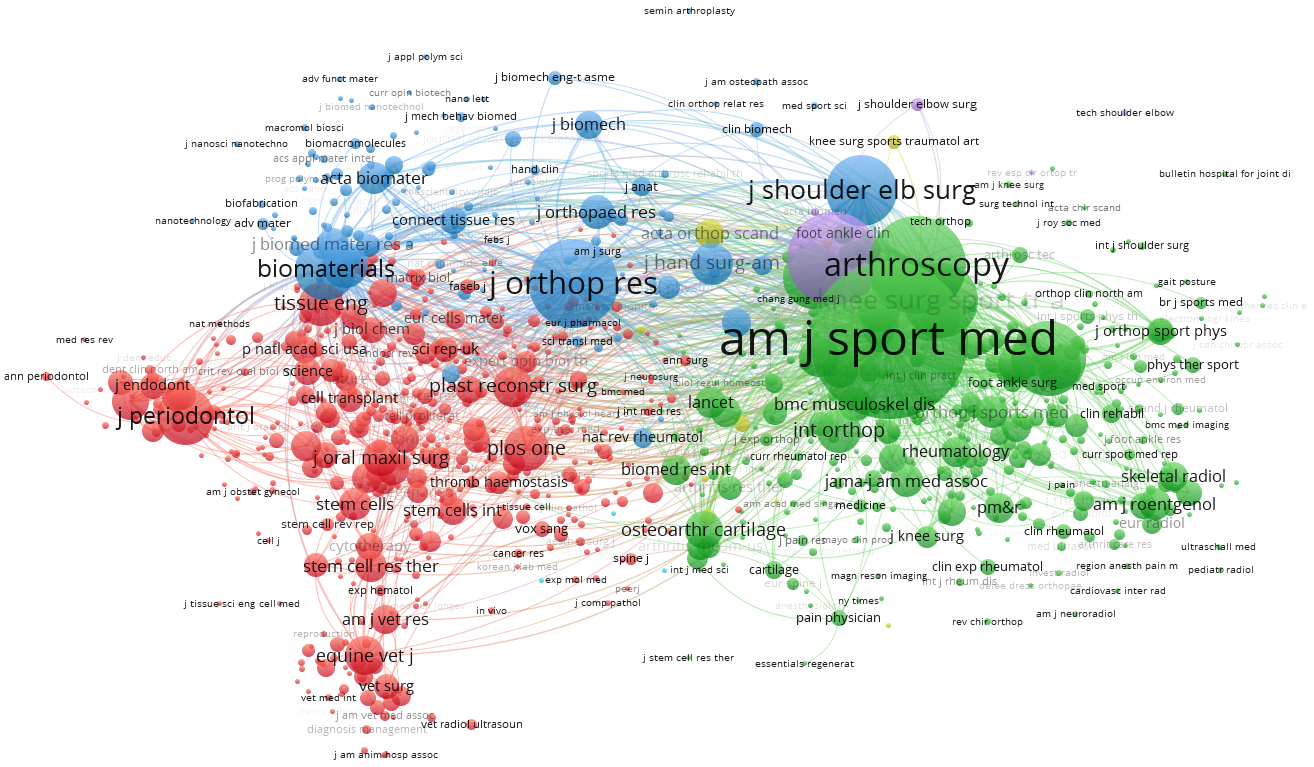

Supplement: Supplementary file 1 [file Datasheet1.zip › Data Sheet 1_v1/Figure/Figure 4(B).jpg]

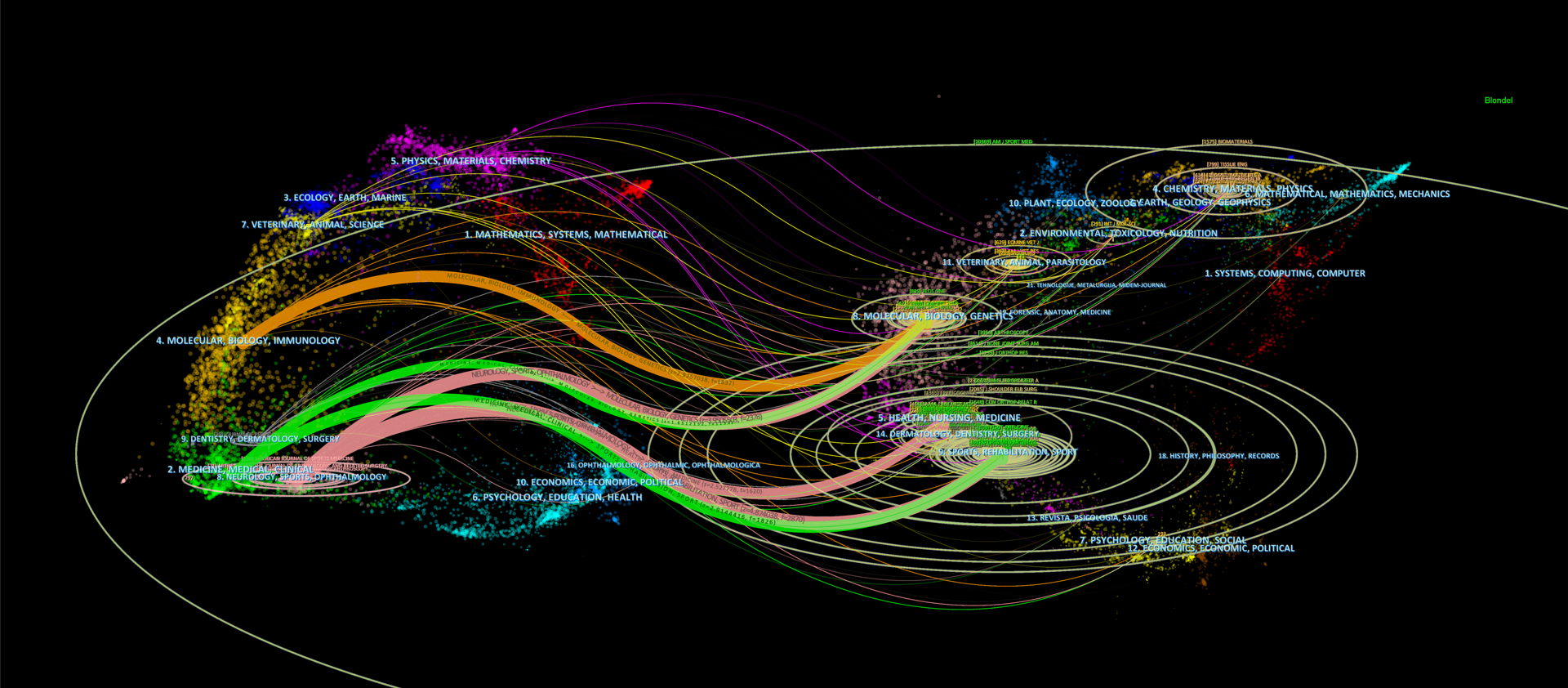

Supplement: Supplementary file 1 [file Datasheet1.zip › Data Sheet 1_v1/Figure/Figure 4(C).jpg]

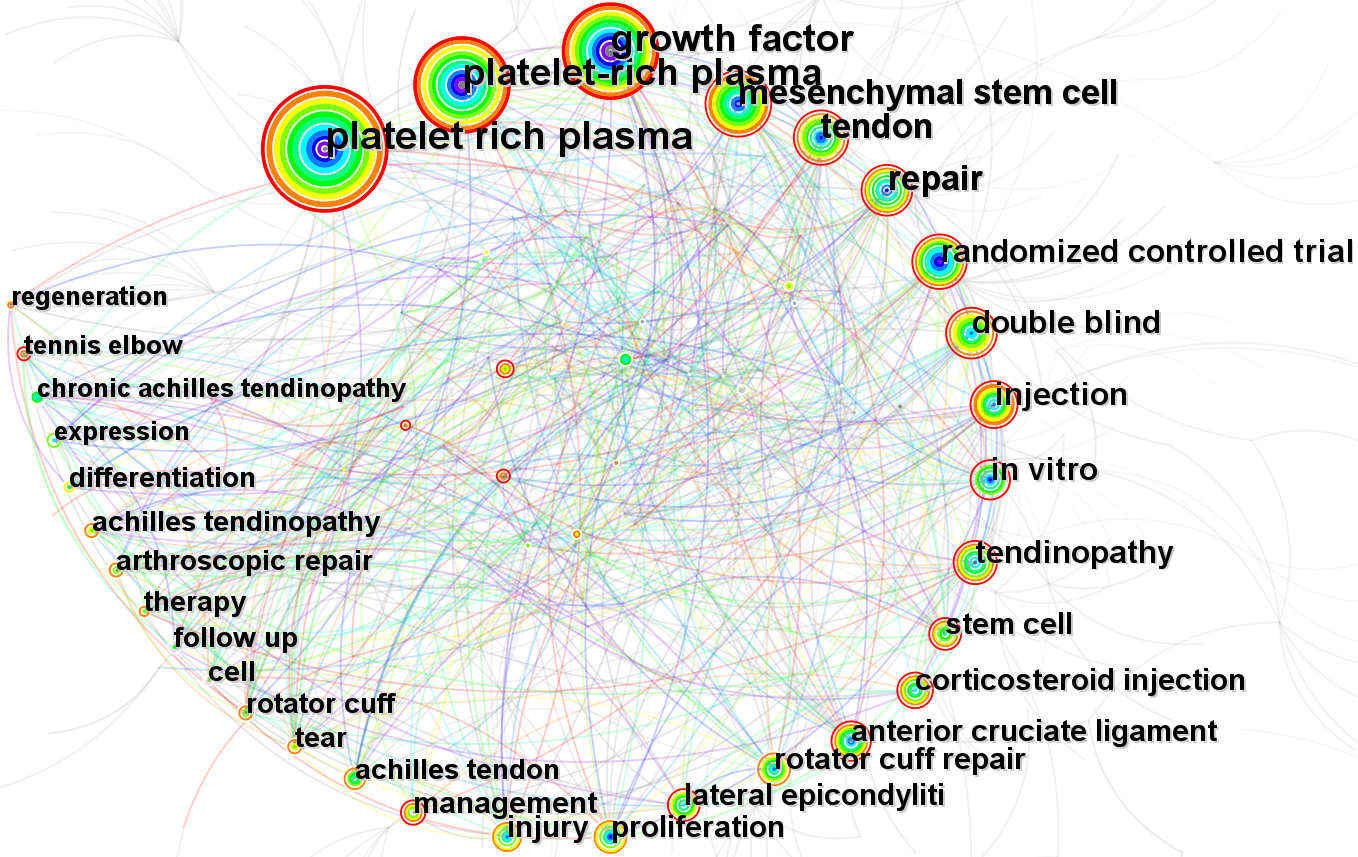

Supplement: Supplementary file 1 [file Datasheet1.zip › Data Sheet 1_v1/Figure/Figure 5(A).jpg]

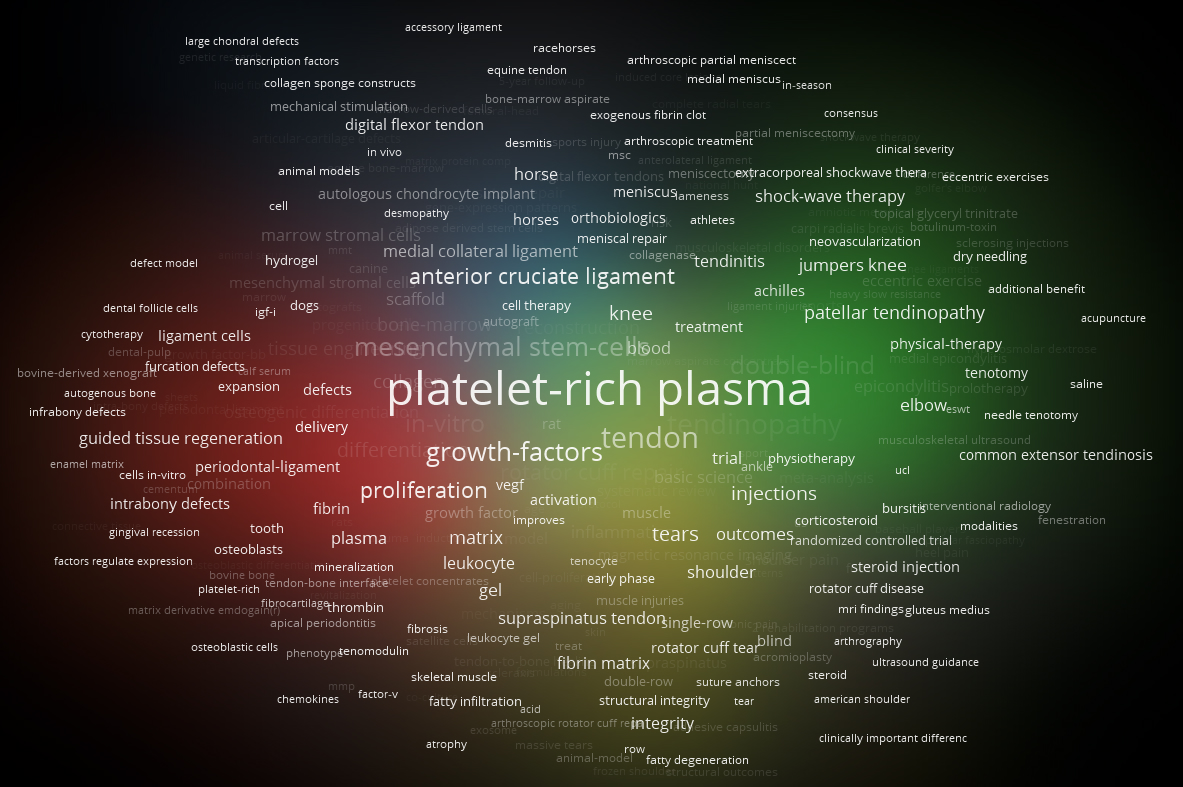

Supplement: Supplementary file 1 [file Datasheet1.zip › Data Sheet 1_v1/Figure/Figure 5(B).jpg]

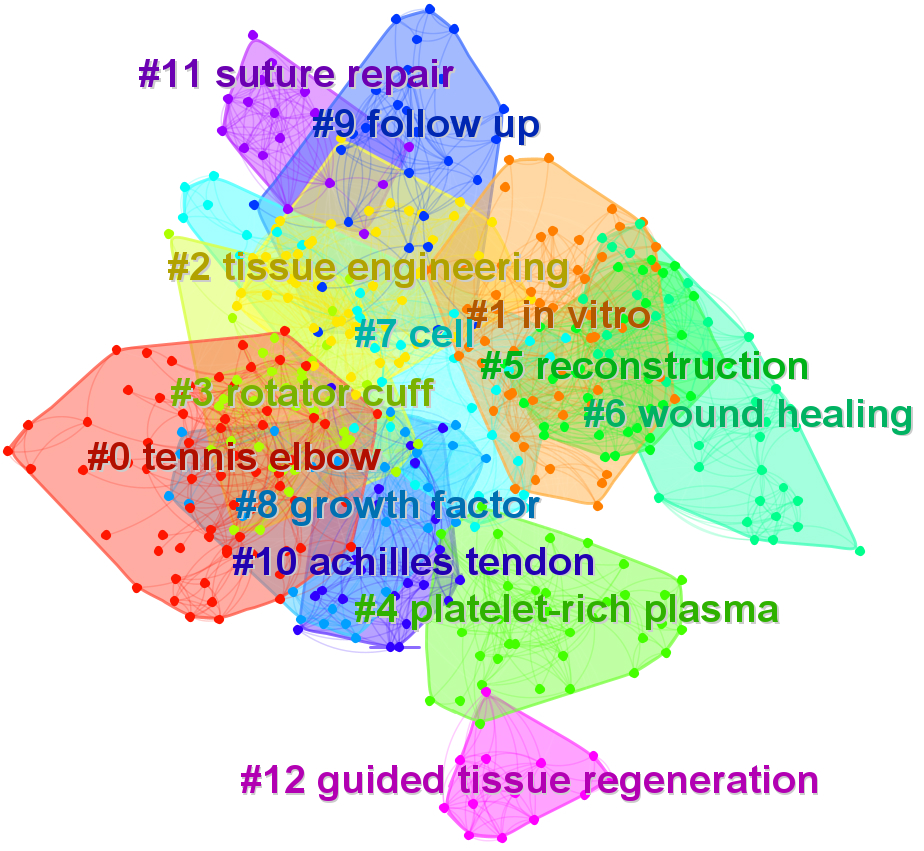

Supplement: Supplementary file 1 [file Datasheet1.zip › Data Sheet 1_v1/Figure/Figure 5(C).jpg]

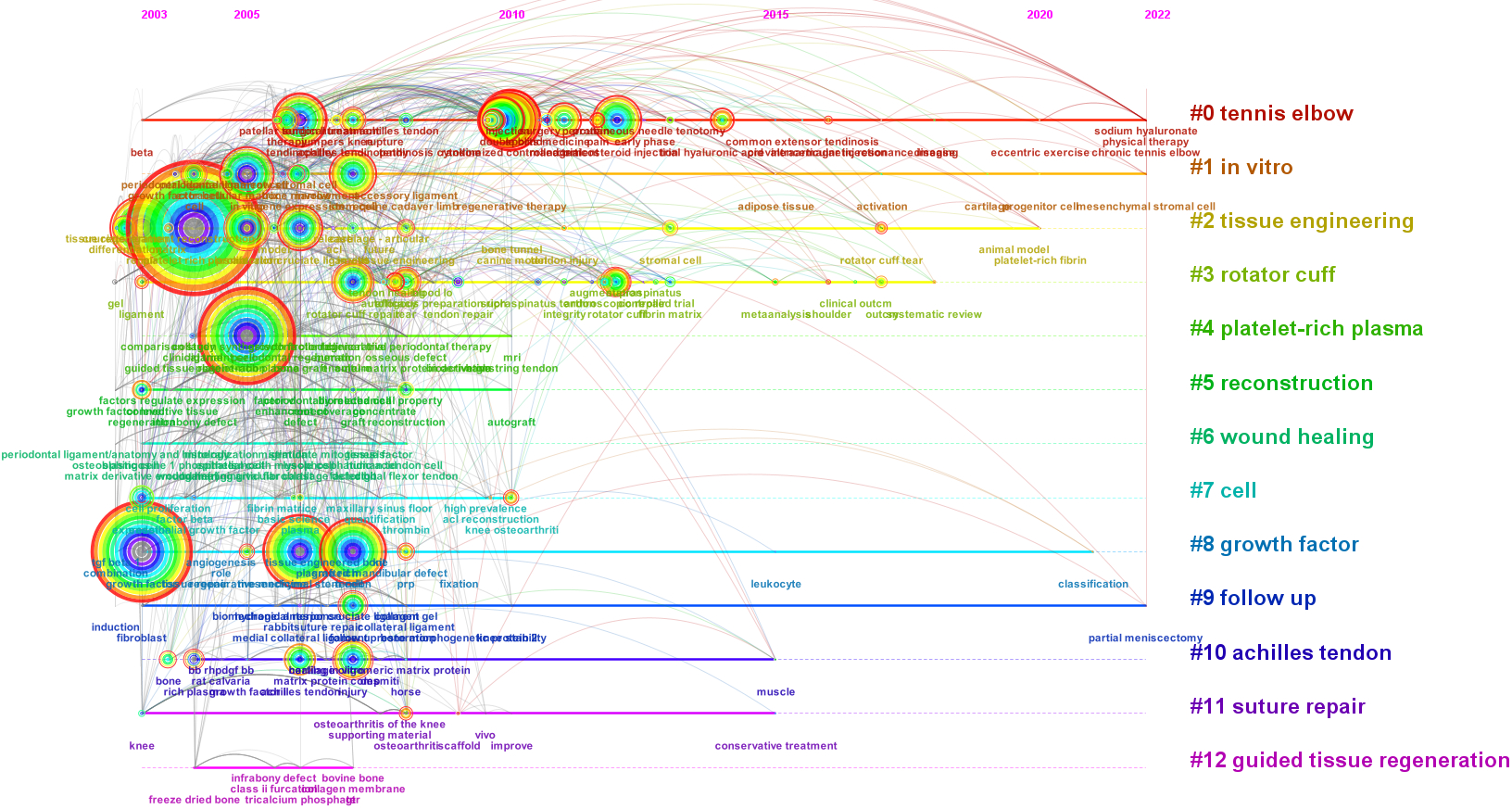

Supplement: Supplementary file 1 [file Datasheet1.zip › Data Sheet 1_v1/Figure/Figure 6.jpg]

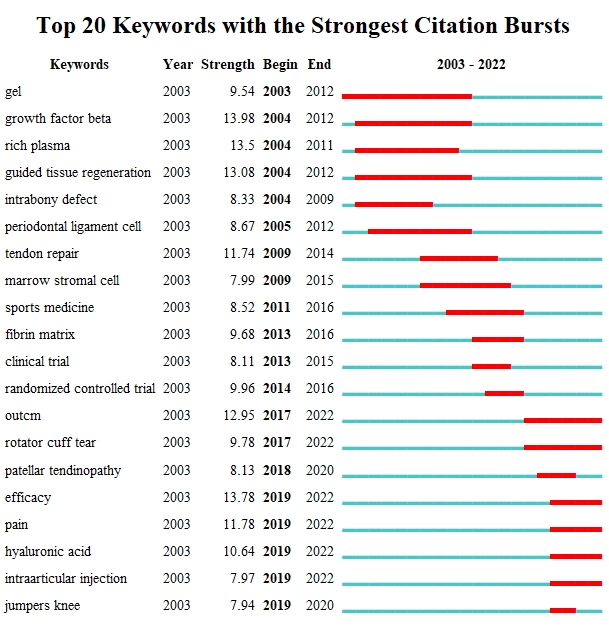

Supplement: Supplementary file 1 [file Datasheet1.zip › Data Sheet 1_v1/Figure/Figure 7(A).jpg]

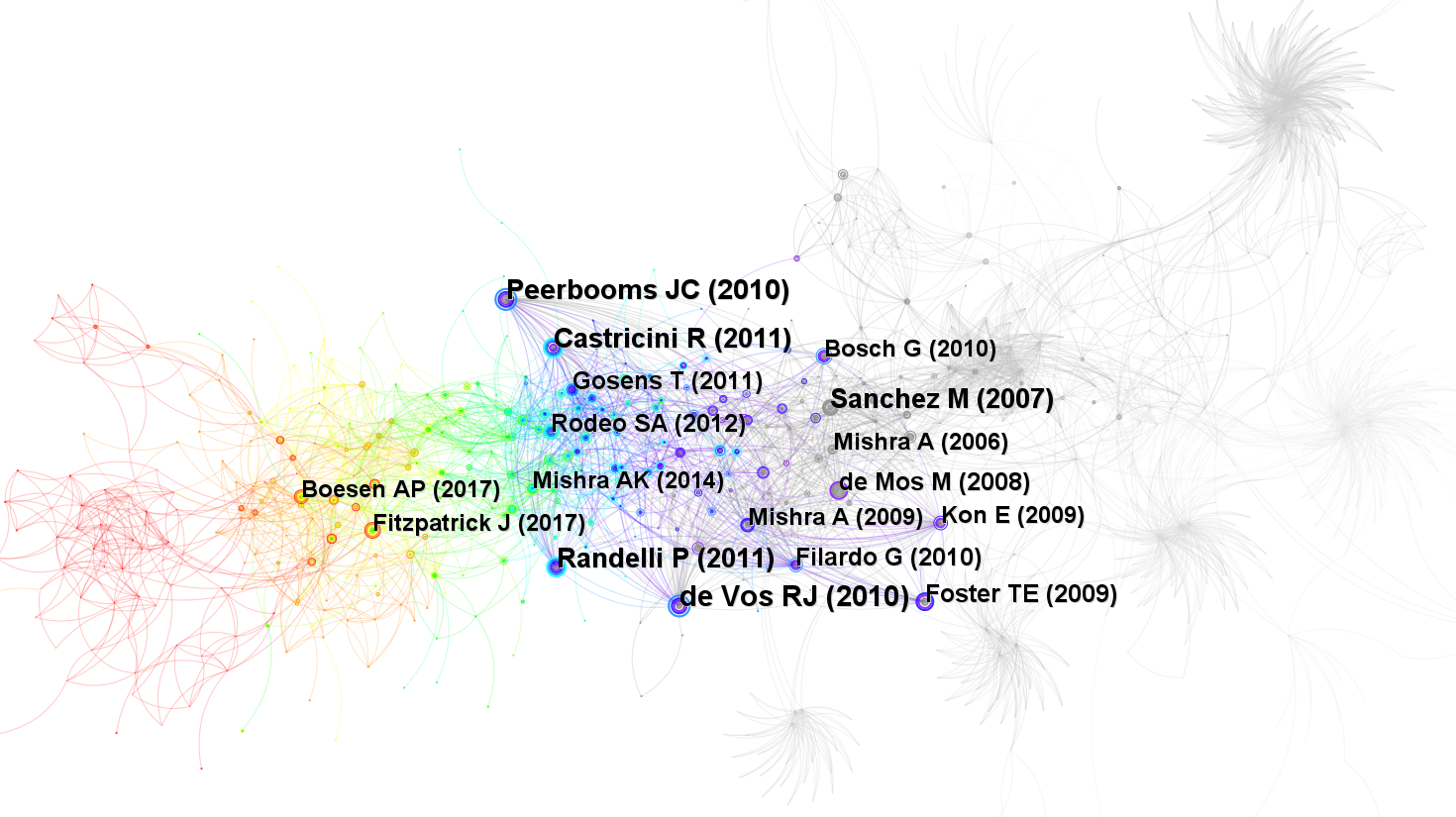

Supplement: Supplementary file 1 [file Datasheet1.zip › Data Sheet 1_v1/Figure/Figure 7(B).jpg]

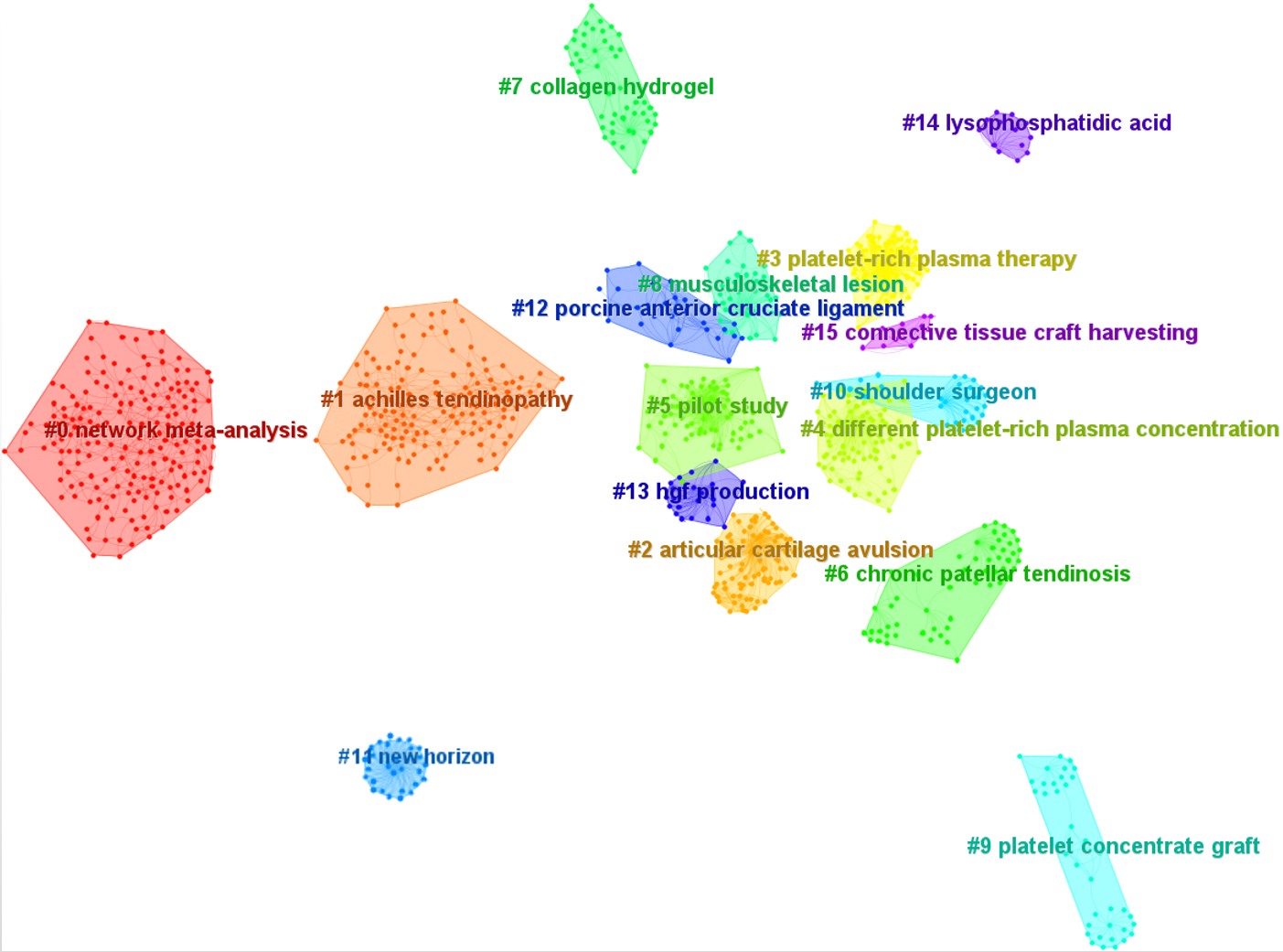

Supplement: Supplementary file 1 [file Datasheet1.zip › Data Sheet 1_v1/Figure/Figure 7(C).jpg]

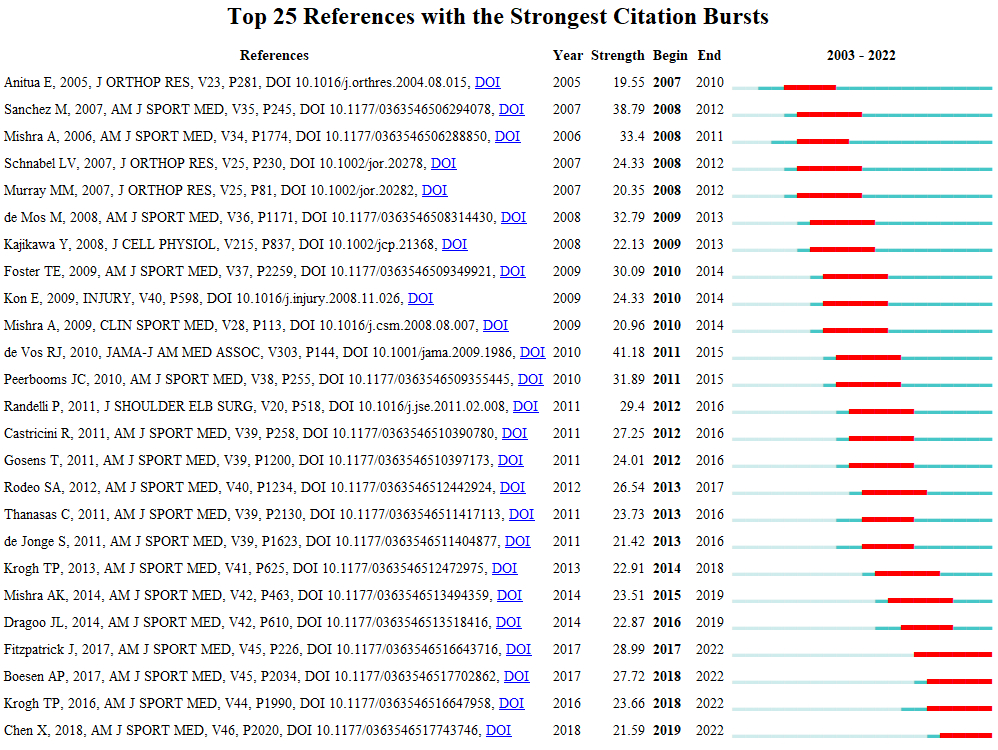

Supplement: Supplementary file 1 [file Datasheet1.zip › Data Sheet 1_v1/Figure/Figure 7(D).jpg]

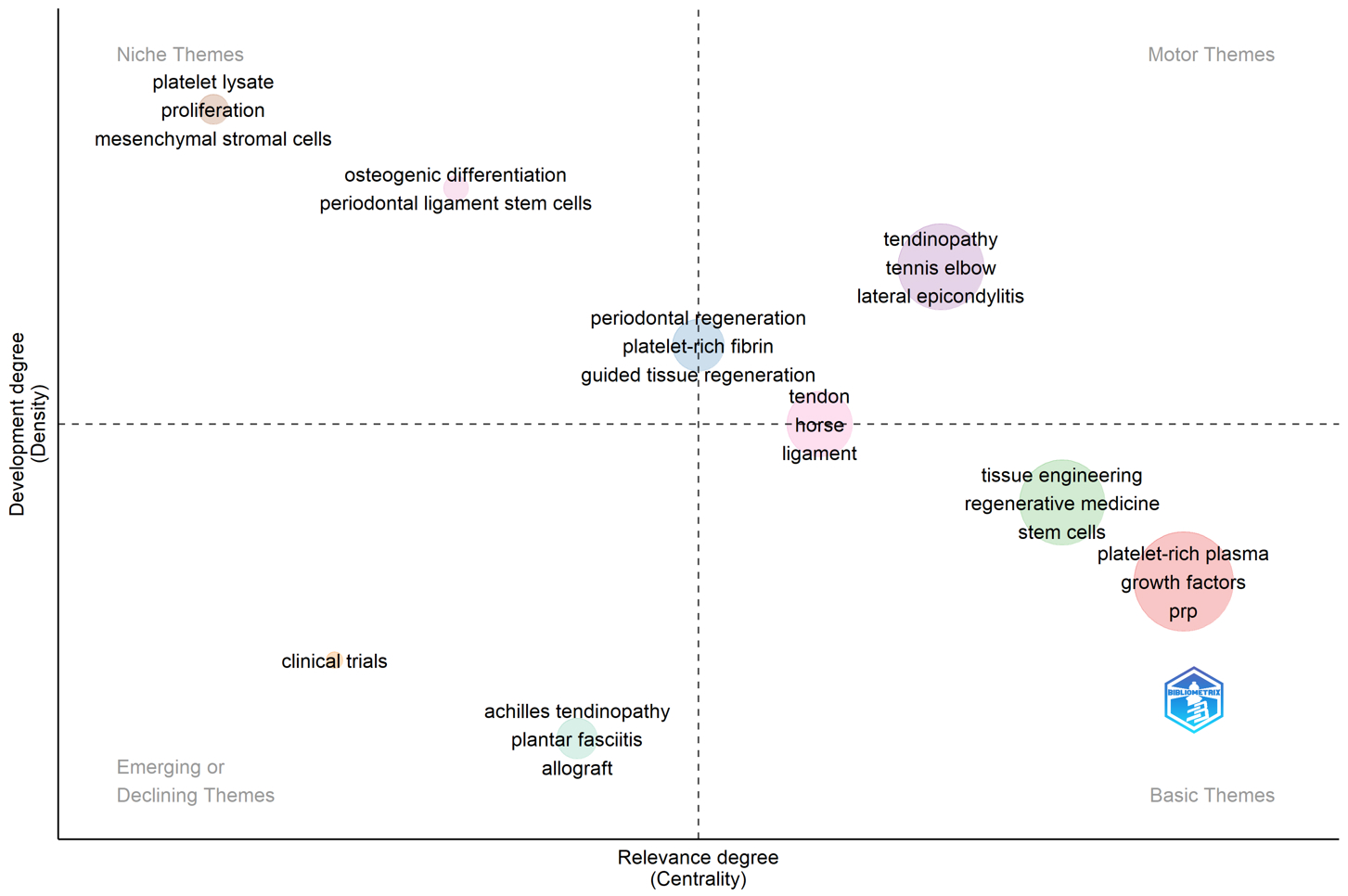

Supplement: Supplementary file 1 [file Datasheet1.zip › Data Sheet 1_v1/Figure/Figure 8.jpg]
